# Deconvolution of the hematopoietic stem cell microenvironment reveals a high degree of specialization and conservation

**DOI:** 10.1016/j.isci.2022.104225

**Published:** 2022-04-08

**Authors:** Jin Ye, Isabel A. Calvo, Itziar Cenzano, Amaia Vilas, Xabier Martinez-de-Morentin, Miren Lasaga, Diego Alignani, Bruno Paiva, Ana C. Viñado, Patxi San Martin-Uriz, Juan P. Romero, Delia Quilez Agreda, Marta Miñana Barrios, Ignacio Sancho-González, Gabriele Todisco, Luca Malcovati, Nuria Planell, Borja Saez, Jesper N. Tegner, Felipe Prosper, David Gomez-Cabrero

**Affiliations:** 1 Bioscience Program, Biological and Environmental Sciences and Engineering Division (BESE), King Abdullah University of Science and Technology KAUST, Thuwal 23955, Saudi Arabia; 2 Universidad de Navarra, CIMA, Hematology-Oncology Program, Instituto de Investigación Sanitaria de Navarra (IdiSNA), 31008 Navarra, Spain; 3 Centro de Investigación Biomédica en Red de Cáncer, CIBERONC, Madrid, Spain; 4 Navarrabiomed, ComplejoHospitalario de Navarra (CHN), Universidad Pública de Navarra (UPNA), IdiSNA, Pamplona, 31008 Navarra, Spain; 5 Hospital Reina Sofía de Tudela, 31500 Navarra, Spain; 6 Department of Molecular Medicine, University of Pavia & Unit of Precision Hematology Oncology, IRCCS S. Matteo Hospital Foundation, 27100 Pavia, Italy; 7 Department of Medicine, Center for Hematology and Regenerative Medicine, Karolinska Institutet, 17177 Stockholm, Sweden; 8 Department of Medicine, Centre for Molecular Medicine, Karolinska Institutet, 17177 Stockholm, Stockholm, Sweden; 9 Computer, Electrical, and Mathematical Sciences and Engineering Division (CEMSE), King Abdullah University of Science and Technology KAUST, Thuwal 23955, Saudi Arabia; 10 Service of Hematology and Cell Therapy, Clínica Universidad de Navarra; CCUN, Pamplona, Navarra, 31008; Spain; 11 Centre for Host Microbiome Interactions, Faculty of Dentistry, Oral & Craniofacial Sciences, King’s College, London WC2R 2LS, UK; 12 Bioengineering Program, Biological and Environmental Sciences and Engineering Division (BESE), King Abdullah University of Science and Technology KAUST, Thuwal 23955, Saudi Arabia

**Keywords:** Biological sciences, Stem cells research, Omics, Transcriptomics

## Abstract

Understanding the regulation of normal and malignant human hematopoiesis requires comprehensive cell atlas of the hematopoietic stem cell (HSC) regulatory microenvironment. Here, we develop a tailored bioinformatic pipeline to integrate public and proprietary single-cell RNA sequencing (scRNA-seq) datasets. As a result, we robustly identify for the first time 14 intermediate cell states and 11 stages of differentiation in the endothelial and mesenchymal BM compartments, respectively. Our data provide the most comprehensive description to date of the murine HSC-regulatory microenvironment and suggest a higher level of specialization of the cellular circuits than previously anticipated. Furthermore, this deep characterization allows inferring conserved features in human, suggesting that the layers of microenvironmental regulation of hematopoiesis may also be shared between species. Our resource and methodology is a stepping-stone toward a comprehensive cell atlas of the BM microenvironment.

## Highlights

• Integration and novel clustering improve the resolution of bone marrow microenvironment
• Novel functional states in endothelium and differentiation stages in the mesenchyme
• Inference of conserved features in human from those identified in mouse

## Graphical abstract

**Figure:**
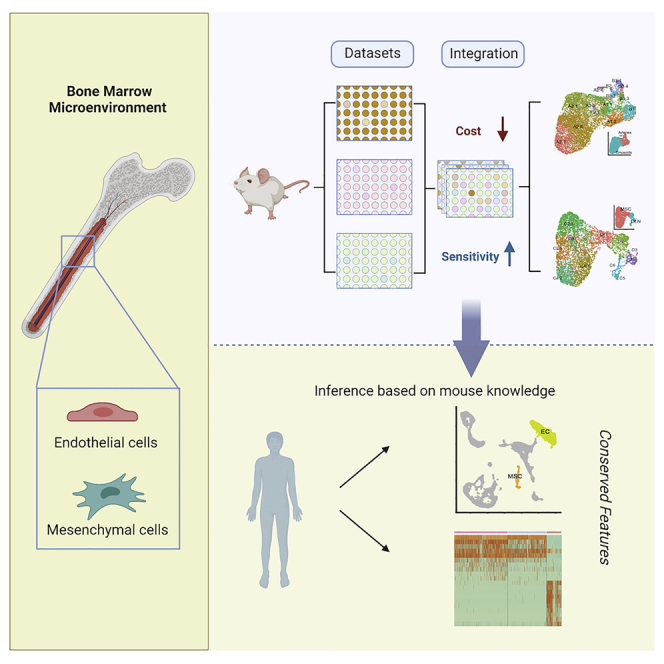

## Introduction

Uncovering pathogenetic mechanisms requires identifying the corresponding major groups of genes in the disease-relevant tissues (Gomez-Cabrero et al., 2014; Wolkenhauer et al., 2013). To this end, collective efforts such as the Human Single-Cell Atlas have been launched, aiming at providing a single-cell map of human tissues and organs. A core case is the hematopoietic system, where single-cell RNA sequencing (scRNA-seq) has allowed to refine our understanding of hematopoiesis in mouse and human (Giladi et al., 2018; Nestorowa et al., 2016; Rodriguez-Fraticelli et al., 2018). Furthermore, these studies have challenged the classical view of hematopoiesis differentiation as a compendium of discrete cellular states with decreased differentiation potential toward a more dynamic view in which hematopoietic stem and progenitor cells (HSPC) gradually pass through a continuum of differentiation states (Karamitros et al., 2017; Laurenti and Göttgens, 2018; Velten et al., 2017; Weinreb et al., 2020). Moreover, recent studies using scRNA-seq technologies have shed light on the organization of the hematopoietic regulatory microenvironment in the mouse (Baccin et al., 2020; Baryawno et al., 2019; Kanazawa et al., 2021; Matsushita et al., 2020; Tikhonova et al., 2019; Wolock et al., 2019; Zhong et al., 2020). These studies have resolved some of the controversies regarding the overlap of stromal populations previously described and the description of certain discrete stromal cells as professional, hematopoietic cytokine-producing populations (Baccin et al., 2020). Moreover, further combination with *in situ* technologies helped to delineate the relationship between specific stromal cell types in the murine bone marrow (BM) (Baccin et al., 2020). This data have provided a wider and more dynamic picture of hematopoiesis and their regulatory microenvironment, allowing for a provocative hypothesis to rise, such as whether their specific association with given niches controls transcriptional states in hematopoietic stem cells and whether these states are reversible upon occupying alternative niches.

Nevertheless, these studies are limited by the number of cells sequenced, potentially hampering our ability to resolve the full spectrum of cellular states and differentiation stages that define the stromal BM microenvironment. More recently, efforts to integrate multiple datasets generated in different labs have been successfully attempted (Dolgalev and Tikhonova, 2021); however, they have failed to provide additional information compared to that derived from the individual studies. Furthermore, knowledge on the conservation of the cellular composition in the human BM stroma is in its infancy due to the difficulty of obtaining high-quality samples with sufficient stromal cell numbers from healthy individuals. This leaves us with two outstanding challenges; how to piece together such different fragments toward a comprehensive molecular atlas and to what extent such an atlas in mice is conserved in the human bone marrow.

Here, we integrate three scRNA-seq datasets (two publicly available (Baryawno et al., 2019; Tikhonova et al., 2019) and one in-house) separately targeting two well-defined populations (endothelial and mesenchymal cells). The integration of distinct datasets required developing tailored bioinformatics pipelines to ensure the robust identification of cell types and stages. We identify 14 endothelial subclusters and 11 subpopulations defining different stages of differentiation in the mesenchyme. Our analysis provides the most comprehensive atlas of the cellular composition in the mouse bone marrow. Last, we asked to what extent such an atlas could provide insight into the less accessible human BM microenvironment. To this end, we made the first pilot study, profiling the human BM using scRNA-seq, which was integrated with our mouse BM atlas. This analysis demonstrated substantial conservation between species.

## Results

### Data integration and high-resolution clustering strategy

**Figure 1:**
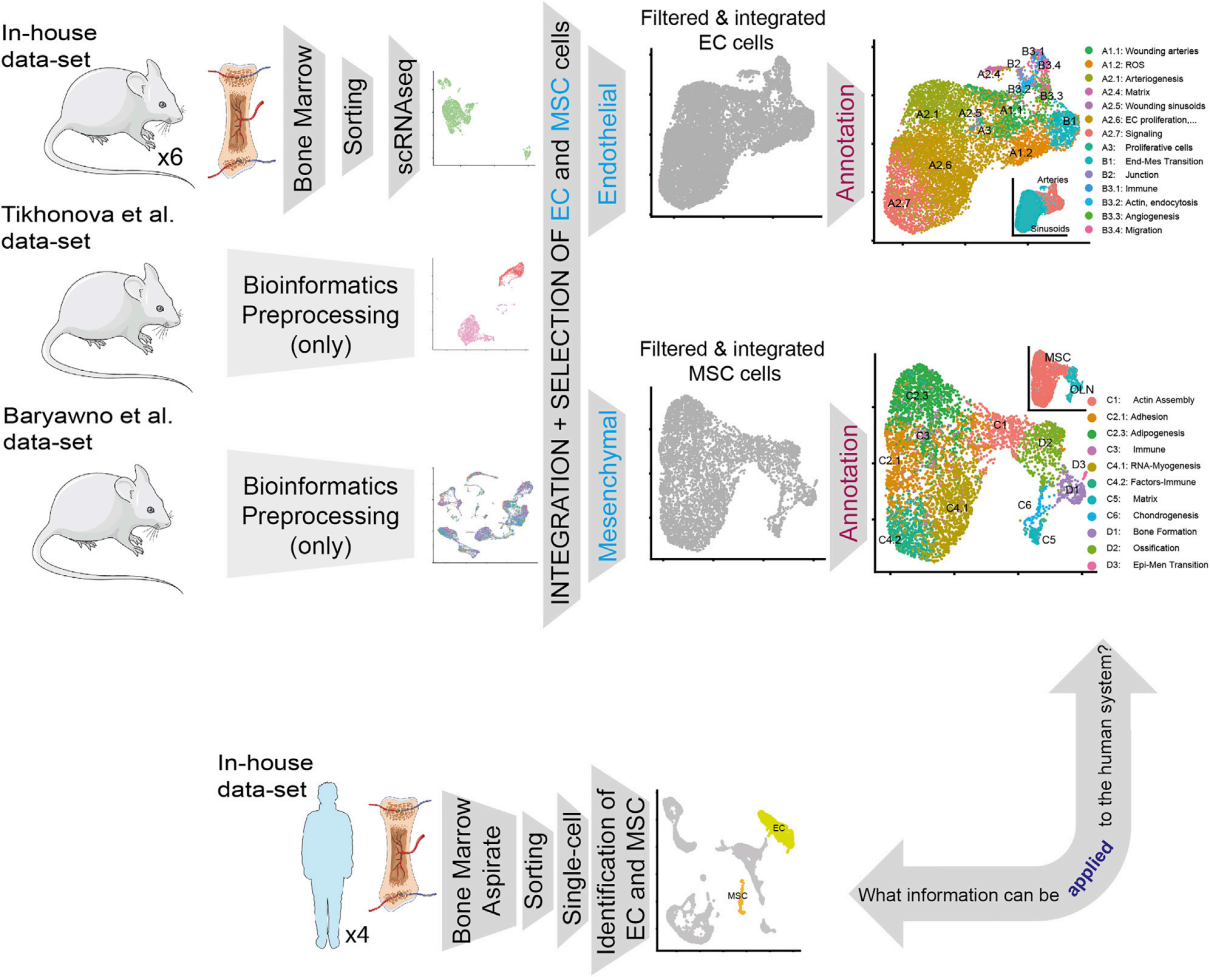
Overview of the paper Graphical brief description of the paper. See also Figure S1.

Figure 1 provides a graphical summary of the experimental design and the analysis flow. We integrated selected subsets of cells from three distinct mouse datasets: two recently published (“Tikhonova et al. (2019), 6626 cells, and “Baryawno et al. (2019), 38443 cells) and an independent dataset (“*In-house*” dataset, 13,402). These datasets differ in the procedures for isolation of cells within the BM microenvironment. This includes unbiased isolation of cells lacking hematopoietic markers (“*Baryawno*” (Baryawno et al., 2019) and “*In-house*”) (Figure S1) versus targeted isolation of populations of interest as in the study by Tikhonova et al. (2019). Furthermore, not every cell type identified in one study is present in the other datasets.

**Figure 2:**
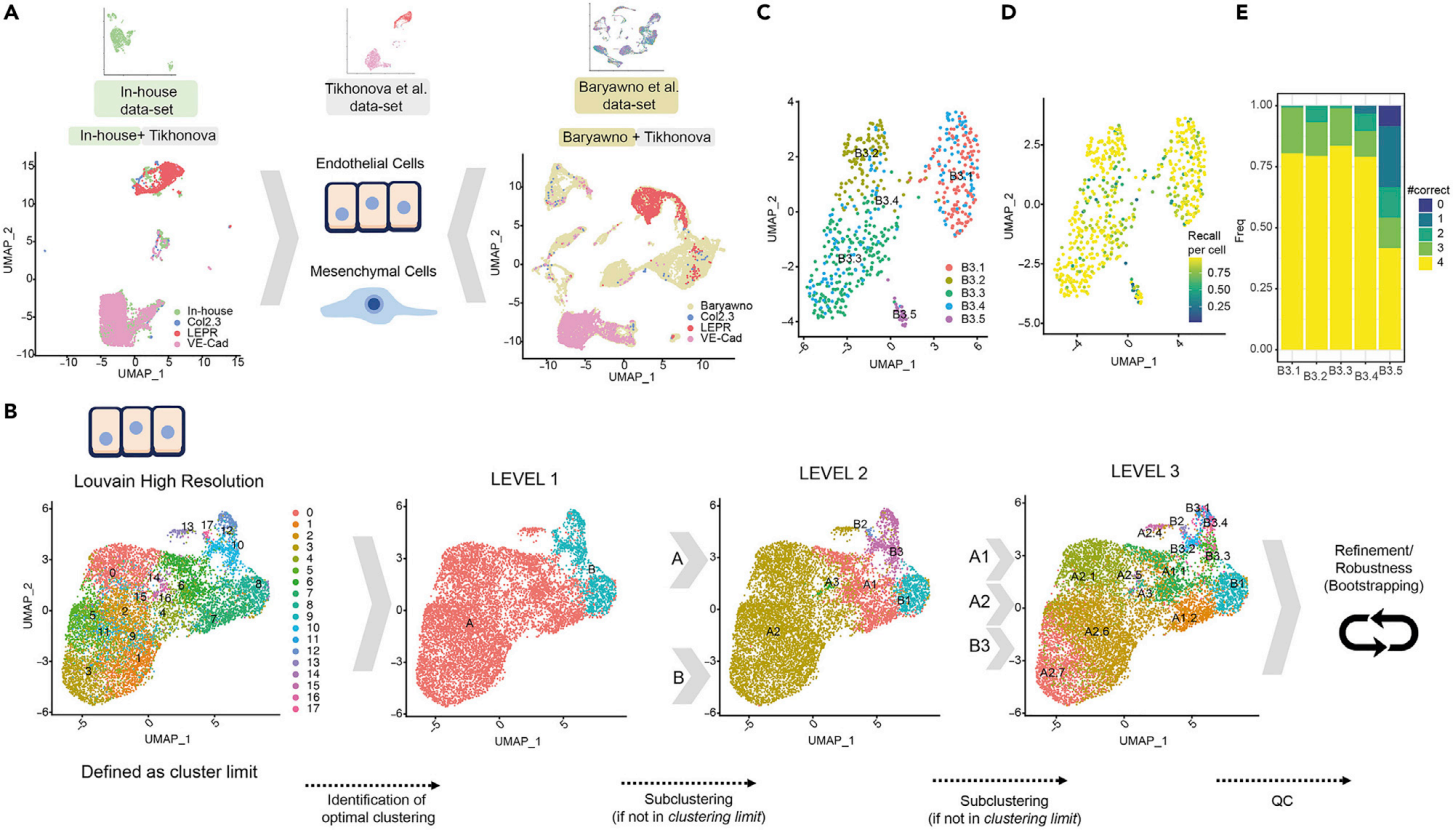
Data integration and high-resolution clustering strategy (A) Integrated analysis of the bone marrow niche datasets (two publicly available, Tikhonova et al., 2019 and Baryawno et al., 2019 and one generated in our lab, *in-house*) separately for two well-defined population (endothelial and mesenchymal cells). Tikhonova et al., 2019 dataset is used as a reference considering their separated cell profiling strategy for COL2.3+, LEPR+, and VE-Cad+ populations. In the top row, a UMAP projection is depicted for each single-cell RNA dataset. In the left-lower (“In-house + Tikhonova”) and in the right-lower (“Baryawno + Tikhonova”), datasets are integrated to identify endothelial and mesenchymal populations. (B) Clustering strategy: the analysis of endothelial cells as an example. An upper limit to the cluster is set for the clustering (left panel) using Louvain high-resolution clustering. Then, an iterative divide-and-conquer strategy identifies the optimal level of clusters at different levels: Level 1 (second panel from the left), Level 2 (third panel), and Level 3 (fourth panel). (C–E) The robustness analysis for sub-clustering B3 (from Level 2 to Level 3). Specifically: (C) subclusters identified, (D) the fraction of assignments to its original cluster *using a random-forest + bootstrapping strategy* and (E) summary of the results (D) per cluster, #correct indicates the times a cluster is a dominant cluster (see STAR Methods) for a cell within it in all pairwise comparisons (see Figure S2 for the sub-clustering analysis of A1 and A2). See also Figures S2 and S3.

We decided to focus our analysis on those bona fide niche populations such as endothelial (EC) and mesenchymal (MSC) cells due to their presence in the three studies and their relevance in controlling hematopoietic stem cell (HSC) maintenance. For the integrative analysis, we used the Tikhonova study as a reference to facilitate the integration, considering that their cells were isolated based on fluorescence reporter expression driven by cell-specific gene promoters: VE-Cad for endothelial cells and LEPR for mesenchymal cells. Therefore, to identify and label the cells of interest, we integrated separately “In house + Tikhonova” and “Baryawno + Tikhonova” (Figure 2A) using the cell labels from the Tikhonova study. As a result, and after quality filters (see STAR Methods), we labeled in each dataset endothelial cells (N = 9587) and mesenchymal cells (N = 5291) that were used for integration of the three datasets for each cell type.

**Figure 3:**
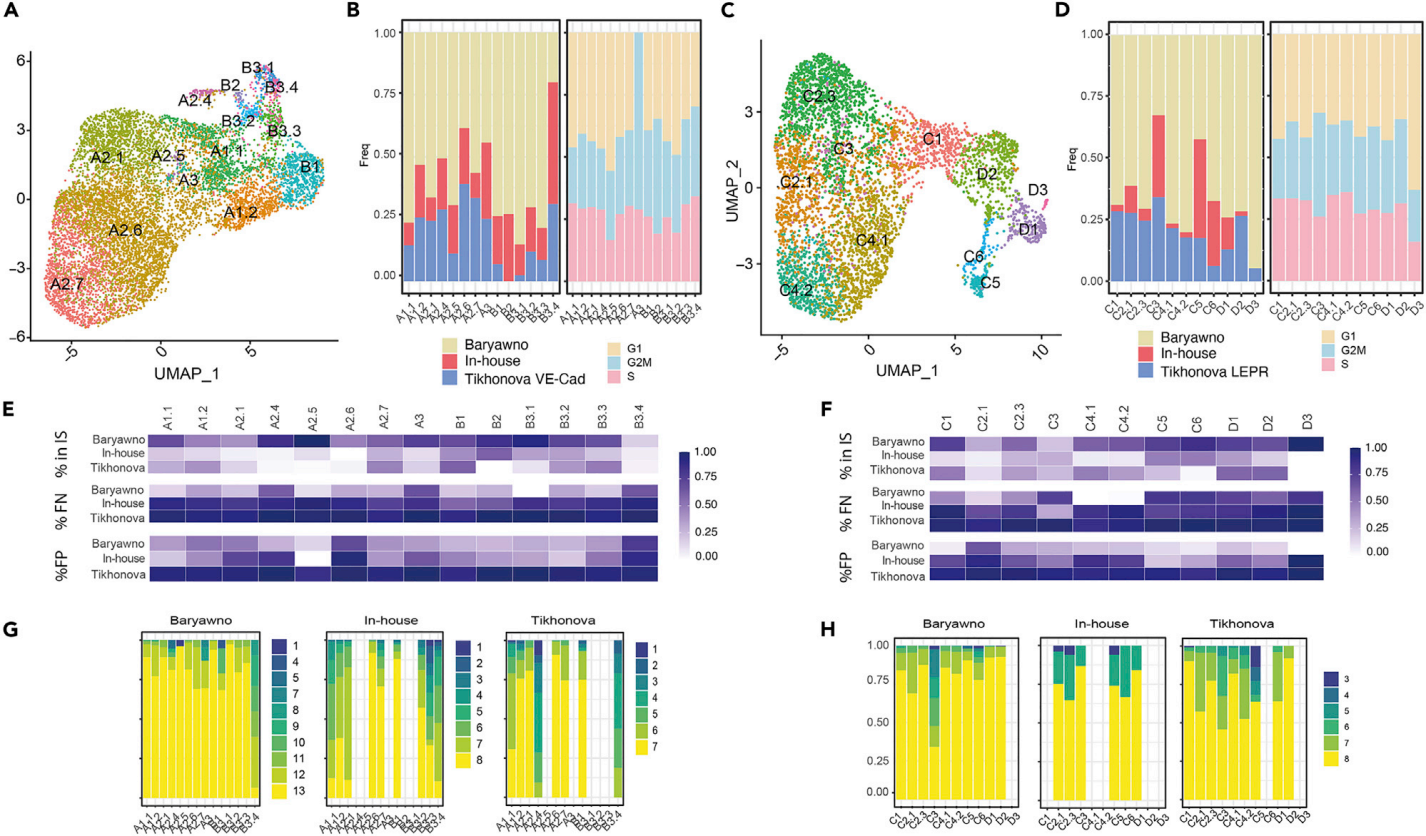
Quality control and added value of the clustering analysis (A) Representation of the final endothelial clusters. (B) Left panel: proportion of cells per dataset in each cluster in the final Endothelial clustering analysis. Right panel: proportion of cells per cell cycle stage using Seurat in each cluster in the final Endothelial clustering analysis. (C and D) Similar as (A and B) for MSC. (E and F) The added value of the integrated approach. Upper panel: every cell depicts the % of markers identified per cluster using only one dataset when compared with the markers identified in the total dataset. Middle panel: every cell depicts the % of False Negatives. Bottom panel: every cell depicts the % of False Positives when comparing the analysis conducted within each dataset with the integrated analysis (considered as the correct result). (E and F) are respectively associated with endothelial and mesenchymal cells. (G and H) Robustness of the cluster characterization using only cells from a single dataset but maintaining the same cluster structure. (G and H) are respectively associated with endothelial and mesenchymal cells. See also Figure S4.

Following the integration, endothelial and mesenchymal groups were used to identify cellular subtypes and stage-specific cells (Figure 2, S2, and S3). However, current state-of-the-art clustering methodologies, including Louvain clustering (Blondel et al., 2008; Traag et al., 2019), could not discriminate robustly among different cell subtypes (Figure 2B left panel and Figure S3A), in part because there is a high degree of cell-to-cell similarity when considering cells of the same origin (Tasic et al., 2016). To enable robust sub-clustering, we customized an existing bootstrapping-based approach. In brief, first, a divide-and-conquer strategy is applied, where the first level of robust clusters is identified (see STAR Methods). Next, we proceeded with another round of clustering, yielding the second level of robust subclusters. As a termination criterion, no sub-clustering was considered if a cluster was found to have no sub-divisions in the Louvain high-resolution clustering (Blondel et al., 2008; Macosko et al., 2015) (Figure 2B left panel and Figure S3A). As a result, the cells are grouped into clusters; then, in the second step, we applied a bootstrapping-based methodology adapted from the Bosiljka study (Tasic et al., 2016) (see STAR Methods and Figure S2A) to quantify the robustness of each cluster. We formulated two evaluation metrics: for each cell, we computed “*how many times it has been correctly assigned to the cluster proposed*” (e.g. recall per cell in Figure S2D), and for each cluster, we quantified “how many times a cluster remains dominant (#Correct) in all comparisons for cells within it” (e.g. #correct in Figure S2E); see STAR Methods for a detailed explanation. If a non-robust cluster was identified, the cells of such cluster were then assigned to the neighboring clusters repeating the random-forest-based strategy. For instance, in the analysis of ECs, three levels of sub-clustering were conducted (Figure 2B middle and right panels). In Level 1, two clusters were identified (A and B), and considering that possible subclusters were identified in the Louvain high-resolution clustering (Figure 2B, (second panel from the left), they were sub-clustered resulting in Level 2. After Level 2, only three subclusters were further investigated (A1, A2, and B3) in Level 3 (Figure 2B, third panel). Level 3 was identified as the final level, and a robustness analysis was conducted for the Level 3 clusters (Figure 2B fourth panel, C-E and Figures S2C-S2H). Non-robust clusters were eliminated and their cells were reassigned to neighboring clusters (see STAR Methods). Similarly, we applied the same robust clustering to the mesenchymal stromal cells (Figures S3A-S3H). By using this approach, we were able to describe 14 subclusters in the endothelium (Figure 2B fourth panel and Figure 3A) and 11 in the mesenchyme (Figure S3A fourth panel and Figure 3C). We observed that both “cell proportions of subclusters in each dataset” and “the cell proportions of the dataset of origin within each subcluster” varied between datasets and subclusters (Figures 3B and 3D, S2B, and S3B). While those biases could be caused for instance by the different selection protocols, we aimed to study the extent of the bias: (a) we did not identify any association with cell stage (Figure 3B and 3D); (b) only clusters B2 and D3 did not have cells from the three datasets. And finally, (c) to quantify the possible biases derived from datasets, we conducted an entropy analysis to evaluate dataset distribution in the clusters; we observed large levels of entropy—varying between clusters—and the averages per cluster are shown in Table S1 (Figure S5).

By integrating the three datasets, distinct cellular states in the microenvironment and the description of the gene markers defining those subtypes were identified. The rationale being that despite the partial overlap observed between the datasets, a larger number of cells would generally contribute to a deeper characterization of the subpopulations. To directly address this and using the markers derived from the integrative analysis, we conducted a series of analyses to assess whether the integration provided additional insights compared to each dataset.

First, we investigated what percentage of final integrated-based markers was recovered by each dataset by itself (Figures 3E and 3F upper panel), what percentage of false negatives (Figures 3E and 3F middle panel) and false positives (Figures 3E and 3F lower panel). For some of the subclusters, such as B3.4, A2.1, and A2.6 (endothelium) or C2.1 and C3 (mesenchyme), over 50% of the markers could not be detected by each dataset separately. In a second analysis, we quantified the robustness of the defined clusters using data from each study separately (Figures 3G and 3H). Only the Baryawno dataset allows for the robust identification of all the subclusters except for B3.4 in the endothelium. However, performing the same clustering strategy using only the Baryawno’s dataset in the endothelium could not identify all the subclusters with the same level of resolution compared to those observed with the integrated dataset (Figure S4). We observed that the marker analysis using several differential expression tools provided similar results (data not shown), demonstrating that our approach is valid even for small size clusters and unbalanced groups.

Next, we used an independent dataset to validate the performance of our integration effort. To that end, we applied our clusters’ signature to derive automatic annotations on an independent dataset (Baccin et al., 2020) using SingleR (Figure S6). This allowed us to discriminate in the independent dataset most of the cellular states we described based on the integration despite the lower number of cells sequenced (Figure S6). These data further validate our integrative approach.

Taken together, these data demonstrate that our customized approach for the integration of multiple datasets allows for a robust deconvolution of cell states when there is a high degree of similarity between cells of the same origin. Furthermore, transcriptional cellular stages inferred from the integration could be applied to further characterize independent single-cell datasets.

### Deep characterization of the BM endothelial cell compartment

**Figure 4:**
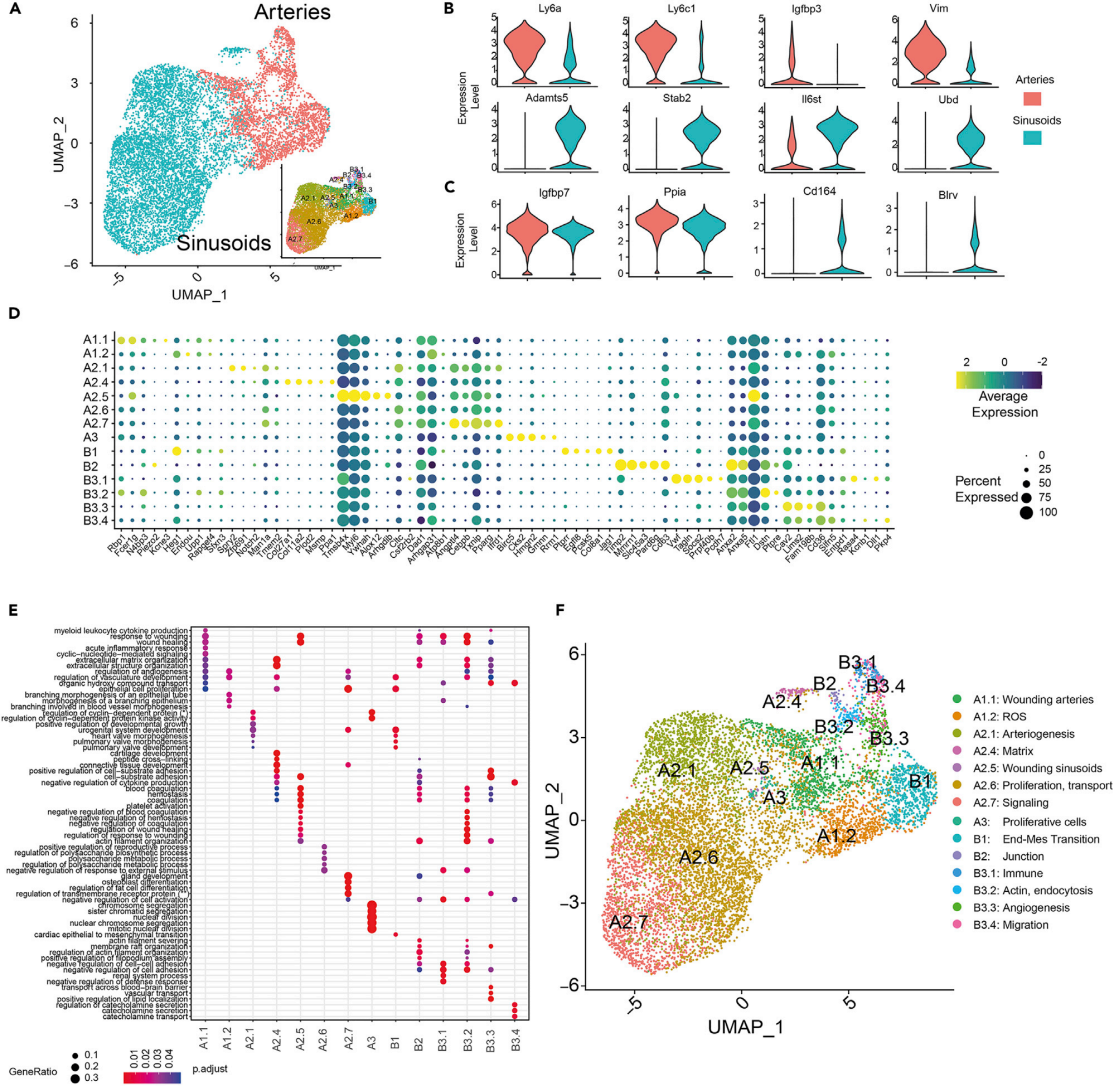
Deep characterization of the endothelial cell compartment in the BM (A) UMAP representation of arteries (red) and sinusoids (blue) within the endothelial cell population. The right-bottom subpanel depicts the final endothelial clusters identified. (B) Violin plot of gene expression for known markers of arteries and sinusoids cell subtypes. (C) Violin plot of gene expression for new marker candidates separating arteries and sinusoids cell subtypes. (D) Dot plot of the top 5 markers for each endothelial subcluster. Dot size corresponds to the proportion of cells within the group expressing each gene, and dot color corresponds to the average expression level. (E) Selected set of gene sets derived from the gene set analysis conducted with top 50 markers per cluster (see STAR Methods). (F) Final clustering of the endothelial cell population and the labeling assigned based on marker genes and gene set analysis. See also Figure S4 and Tables S2, S3, and S4.

Next, we aimed to characterize each of those 14 endothelial subclusters (Figure 3A) based on the identified markers (Table S2). Using the expression of those molecular markers, we could discriminate between arteries and sinusoids (Figure 4A and Table S3) in agreement with previous reports (Hooper et al., 2009; Itkin et al., 2016; Rafii et al., 2016). Arterial clusters showed high expression of specific arterial genes such as *Ly6a, Ly6c1, Igfbp3,* and *Vim* (Figure 4B upper panels). At the same time, sinusoidal cells were defined by their characteristic signature expressing *Adamts5, Stab2, Il6st, and Ubd* (Figure 4B lower panels). Importantly, besides these already known markers, differential expression analysis of the integrative datasets revealed some novel genes specific of each endothelial population, such as *Igfbp7* and *Ppia* for arteries and *Cd164* or *Blrv* for sinusoids (Figure 4C). The expression of these genes would be consistent with the role *Igfbp7* and *Blvrb* in the maintenance of endothelial vasculature homeostasis (Klóska et al., 2019; Tamura et al., 2009).

Beyond characterizing arteries and sinusoids, we annotated their respective cell states using cluster’s markers based on genes (Table S2, Figure 4D) and gene sets (derived from gene set enrichment analysis, see STAR Methods for a detailed version of the annotation; Table S4, Figure 4E). Our final annotation described in Figure 4F was based in conserved Gene Ontology terms, and supported, with no contradictory gene sets, from Reactome and KEGG pathways analysis (Table S4/data not shown). Gene sets related to vasculature development and remodeling were identified within the endothelial subclusters, confirming the identity of this cell population (Table S4). We uncovered two subclusters (A1.1 and A2.5, respectively) within the arteries and sinusoids groups, which were enriched in gene sets involved in wounding. This finding is in agreement with the role of EC in pro- and antithrombotic processes (Yau et al., 2015). Gene sets involved in extracellular matrix assembly, cell adhesion, and migration processes were specifically enriched in arteries, in line with the importance of these biological processes for vascular morphogenesis (Davis and Senger, 2005). In relation to the structural support provided by arterial cells, subcluster B3.2 (actin, endocytosis) implicated in matrix remodeling was defined by the expression of *RhoC, Apln,* and *Anxa2*. Other arterial subclusters such as ROS and Immune (A1.2 and B3.1, respectively) include highly expressed gene sets involved in the regulation of reactive oxygen species metabolic process and cytokine-mediated signaling pathway. These findings are in line with the role of ECs in maintaining the redox balance and leukocyte regulation (Testa et al., 2016; Zhao et al., 2012). Sinusoidal-endothelial subclusters such as A2.1 and A2.6 showed enrichment in GO terms related to artery development and endothelial proliferation processes, two critical steps within the process of angiogenesis (Naito et al., 2021). Furthermore, the sinusoidal subcluster A2.7 expressed gene sets involved in ion transport and signaling-related signatures. This is in concordance with the need of EC to constantly sense and adapt to alterations in response to microenvironmental cues (March et al., 2009; Quillon et al., 2015). Of note, ion channels play a role in EC functions controlled by intracellular Ca^2+^ signals, such as the production and release of many vasoactive factors such as nitric oxide. In addition, these channels are involved in the regulation of the traffic of macromolecules, controlling intercellular permeability, EC proliferation, and angiogenesis (Nilius and Droogmans, 2001). Importantly, several markers that were found only with the dataset integration correspond to genes within the GO categories used to label the clusters, hence, revealing their important role in defining the function of these cell states. For example, in subcluster B2, genes such as *Gja1*, *Tgfb3,* and *Ablim2* are involved in regulating cell junctions and cytoskeletal organization (Barrientos et al., 2007; Okamoto and Suzuki, 2017).

Taken together, these results suggest a previously unrecognized level of specialization of the bone marrow endothelial cells. Furthermore, the specificity of the distinct functional states in the EC suggest that the endothelial compartment is a more dynamic and flexible tissue with a richer intrinsic repertoire than previously anticipated. However, functional validation is required to confirm the putative role of each EC intermediate cell state described here.

### Deep characterization of the BM mesenchymal cell compartment

**Figure 5:**
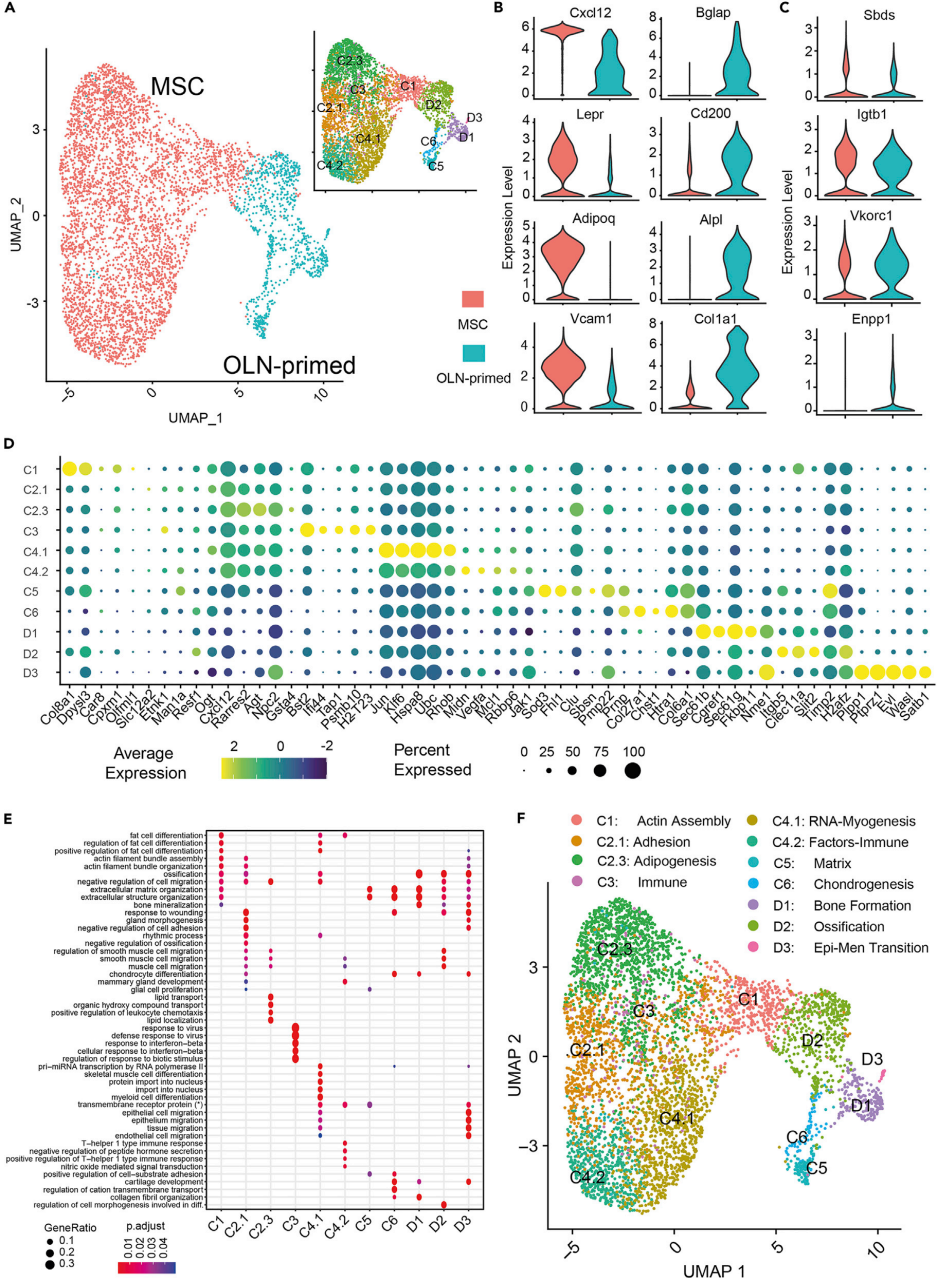
Deep characterization of the mesenchymal cell compartment in the BM (A) UMAP representation of mesenchymal (red) and osteolineage-primed (OLN-primed) (blue) within the mesenchymal compartment. The right-upper subpanel depicts the final mesenchymal clusters identified. (B) Violin plot of gene expression for known markers of mesenchymal (red) and osteolineage-primed (blue) cells. (C) Violin plot of gene expression for new marker candidates separating mesenchymal (red) and osteolineage primed (blue) cells. (D) Dot plot of the top 5 markers for each mesenchymal subcluster. Dot size corresponds to the proportion of cells within the group expressing each gene, and dot color corresponds to the average expression level. (E) Selected set of gene sets derived from the gene set analysis conducted with top 50 markers per cluster (see STAR Methods). (∗) transmembrane receptor protein serine/threonine kinase signaling pathway. (F) Final clustering of the mesenchymal cell population and the annotation based on marker genes and gene set analysis. See also Figure S4 and Tables S5, S6 and S7.

Applying the same robust clustering to mesenchymal stromal cells, we identified 11 subclusters and proceeded with the annotation (Figure 3C and S3, and Tables S5, S6, and S7). Based on the expression of canonical markers, we first discriminated clusters between early mesenchymal (MSC), and cells already committed to the osteolineage (OLN-primed) (Figure 5A and Table S6). The high expression of *Cxcl12*, *LepR*, *Adipoq,* or *Vcam1,* among others, confirmed the identity of the early MSC group (Figure 5B left column); whereas *Bglap*, *Cd200*, *Alpl,* or *Col1a1* expression revealed the presence of osteolineage committed cells within the mesenchymal compartment (Figure 5B right column). Furthermore, we identified a number of previously unrecognized, differentially expressed genes between the MSC and OLN-primed clusters such as *Sbds* and *Itgb1* for MSCs and *Enpp1* and *Vkorc1*, for OLN-primed cell type population (Figure 5C). *Itgb1*, highly expressed in MSC is implicated in human chondrogenic differentiation of mesenchymal cells (Hamidouche et al., 2009). Among OLN-primed specific markers, *Enpp1* and *Vkorc1* have been shown to regulate bone development by regulating bone calcification (Hajjawi et al., 2014; Mackenzie et al., 2012; Price, 1985; Spohn et al., 2009).

Besides MSCs and OLN-primed MSCs, we identified additional subpopulations. Through the marker identification and gene set analysis of the 11 subclusters (Figures 5D and 5E and Tables S5, S6, and S7) we were able to characterize and label each of the clusters as shown in Figure 5F. GO terms such as adipogenesis, assembly and organization, immune response, cell migration, or muscle differentiation were enriched in the C2.3, C2.1, C1, C3, C4.2, and C4.1 subsets respectively, confirming the identity of this MSC cell group. Furthermore, terms related to extracellular matrix, chondrocyte differentiation, and bone development, including bone formation, ossification, or epithelial migration, among others, were identified in the OLN-primed subclusters (C5, C6, and D, respectively), verifying the identity of these more mature cells (OLN-primed cells) within the mesenchymal stromal cells.

Taken together, these results demonstrate that the newly identified mesenchymal subpopulations could not be properly characterized without the multi-dataset integration and a novel clustering approach. Further, our data provide evidence of the heterogeneity of the mesenchymal compartment in the BM.

### Composition of the human endothelial and mesenchymal BM microenvironment

**Figure 6:**
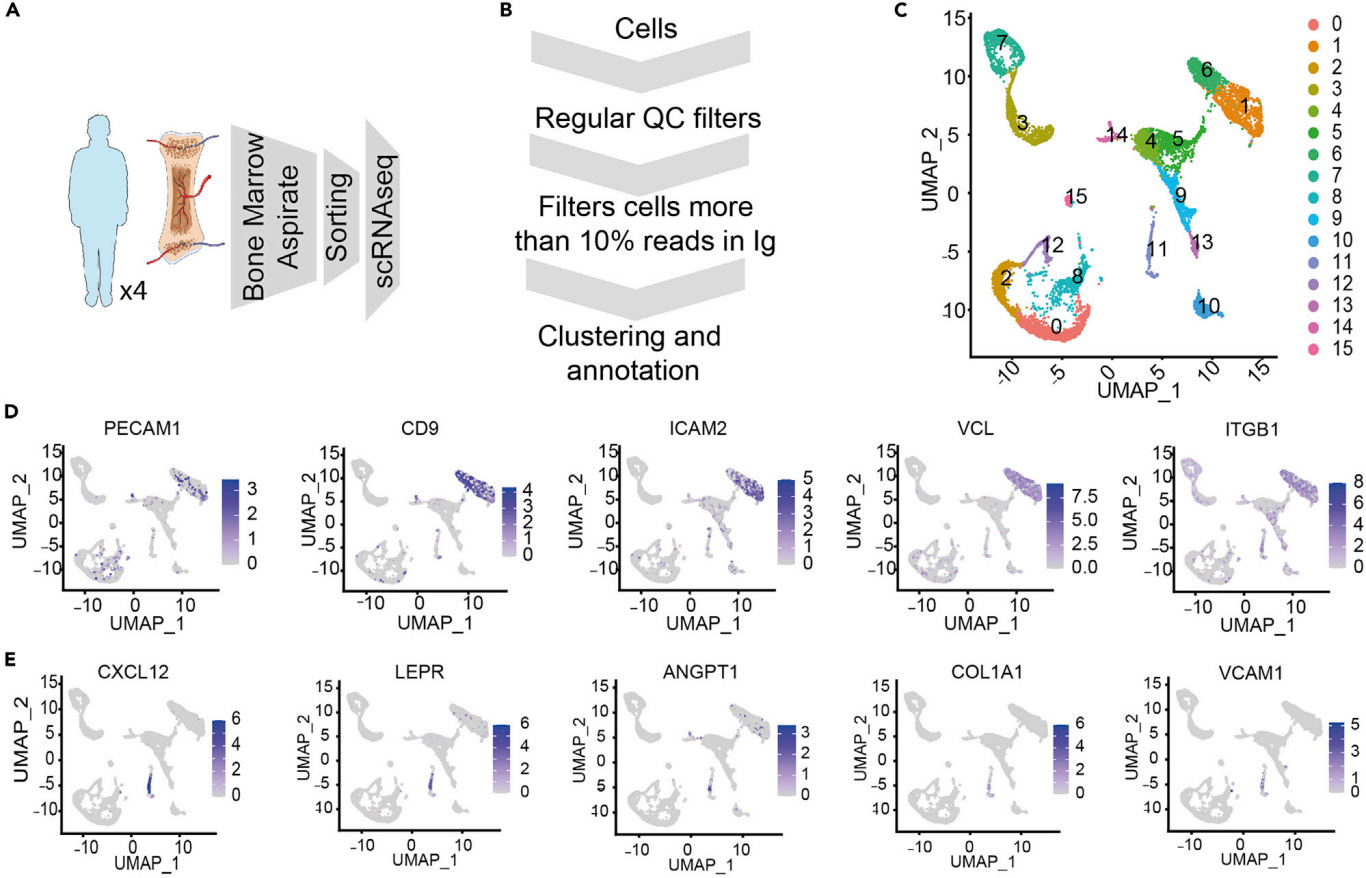
Composition of the human endothelial and mesenchymal BM microenvironment (A) Experimental design for the human BMM characterization. (B) Scheme of customized bioinformatics pipeline filtering the cells with a large number of Immunoglobulin genes. (C) UMAP visualization of color-coded clustering of the human BM microenvironment after filtering cells. (D and E) Expression of representative markers for endothelial population (D) and mesenchymal-osteolineage cells (E) using an UMAP representation. See also Figure S5 and Tables S8 and S9.

While our data reveal a previously unrecognized heterogeneity in the murine BM endothelial and mesenchymal compartments, information about the composition of the human microenvironment and how much of this heterogeneity is observed in humans remains unanswered. To address this issue, we performed scRNA-seq analysis in prospectively isolated EC (TO-PRO-3^-^, CD45^−^, CD235^-^, Lin^−^, CD31^+^, and CD9^+^(Barreiro et al., 2005; Kenswil et al., 2018)) and MSC-OLN (TO-PRO-3^-^, CD45^−^, CD235^-^,Lin^−^,CD271^+^(Ghazanfari et al., 2016; Hashemi et al., 1991; Quirici et al., 2002), and CD146^+/−^) (Figure S7A) from iliac crest BM aspirates from four healthy young adults (20–30 years of age) (Figure 6A). As described in Figure 6B, we added additional filtering steps in the bioinformatic analysis to identify the two populations of interest, EC and MSC. As an additional quality control, we estimated the contribution of each human sample to EC and MSC subsets and cell cycle status (Figures S7B and S7C). The EC (907 and 658 cells, clusters 1 and 6, respectively) (Figures 6C and S7B middle panel) identity was confirmed based on the expression of canonical endothelial markers such as *PECAM1* (coding for *CD31*)*, CD9, ICAM2, VLC,* and *ITGB1* (Figure 6D and Table S8). In addition, examining functional pathways in clusters 1 and 6 revealed enrichment in GO terms associated with blood coagulation and hemostasis **(**Table S9). The MSC identity (249 cells, cluster 11, Figures 6C and S7B middle panel) was confirmed by the expression of the mesenchymal specific genes (*CXCL12 and LEPR*) and the OLN-primed specific genes *ANGPT1, COL1A1, and VCAM1,* among others (Figure 6E and Table S8). Furthermore, enrichment in functions associated with extracellular matrix organization and response to the mechanical stimulus was demonstrated in osteolineage cells (Table S9). In summary, the generated human data suggest that single-cell RNA sequencing from iliac crest aspirates can aid in describing the complexity of the human BM microenvironment. Nevertheless, the limited number of EC and MSC, as well as the presence of contaminating populations did not allow a fine-grained clustering as the one performed in the mouse data.

### Substantial conservation of the EC and MSC population in the human BM microenvironment

Based on the limitations of the human data, we next investigated to what extent the knowledge uncovered in mouse could be applied to identify subpopulations/cell states in the human BM microenvironment. As a first step, we used single-cell mouse data to annotate the human cells using SingleR (Aran et al., 2019), separately for EC (Figures S8A and S8B) and MSC (Figures S8C and S8D). We observed that MVG identified in mouse allowed us to separate the cells into clusters for both human EC and MSC (Figures S6A–S6D). As a result, this analysis suggests that part of the biological mechanisms defining the BM microenvironment may be shared between species.

**Figure 7:**
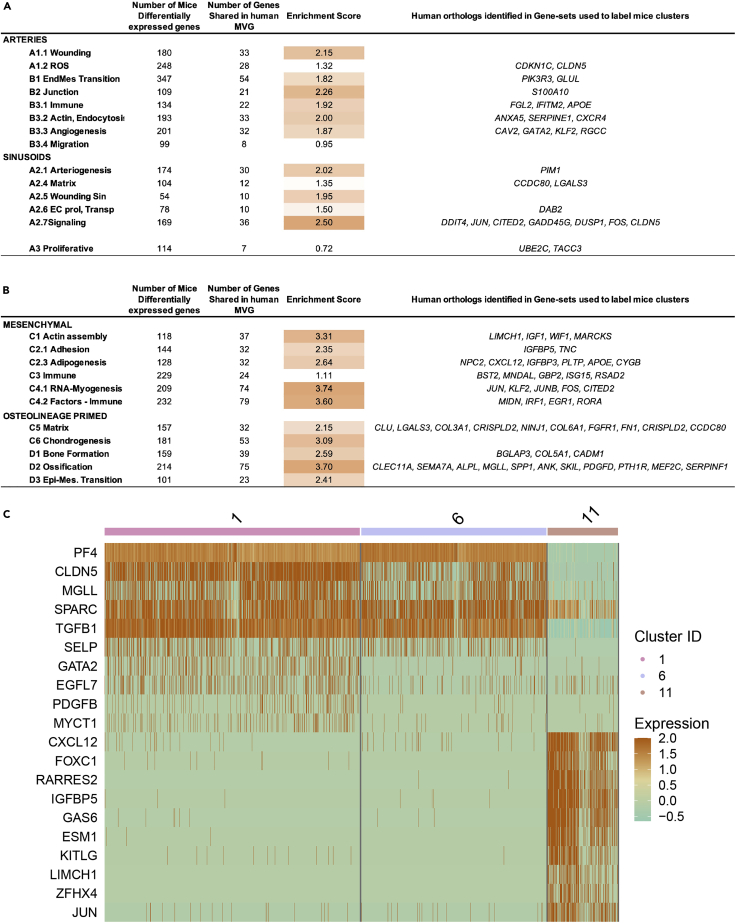
Conservation analysis of the EC and MSC population in the human BM microenvironment (A and B) Quantification of the conservation for EC (A) and MSC-OLN (B) cells for each cluster. The enrichment of those genes that are cluster markers in mouse and observed in most variable genes (MVG) of EC and MSC human cells, respectively. The right column shows, among the genes identified in human, those that are part of the gene sets used to label the cluster. See also Figure S6 and Tables S10 and S11. (C) Heatmap of the cytokines, secreted molecules, and growth factors in EC and MSC.

Therefore, we decided to investigate the enrichment of conserved features (genes) between mouse and human; therefore, an enrichment score (ES) was computed for each cluster for EC and MSC separately (see STAR Methods) (Figure 7A and Tables S10 and S11). In the case of endothelial cells, the enrichment score was up to 2-fold (Figure 7A): wounding (A1.1) with 2.15-ES, the junction (B2) 2.26-ES, arteriogenesis (A2.1) 2.02-ES, and signaling (A2.7) 2.5-ES). Importantly, for some of the subclusters as sinusoidal signaling (A2.7) and the arterial of angiogenesis (B3.3), these shared genes are critical for defining each of those EC functional states (Table S10). Both human and mouse ECs express *DDIT4*, *JUN*, *CITED2*, *GADD45G*, *DUSP1*, *FOS,* and *CLDN5,* which are part of a wide array of transcription factors, growth factors, and signaling pathways that have been described to regulate the maintenance of vascular homeostasis under physiological conditions (Echavarria and Hussain, 2013; Escudero-Esparza et al., 2012; Jia et al., 2016). Similarly, ECs subclusters involved in angiogenesis in both species shared the expression of *RGCC*, *GATA2*, *KLF2,* and *CAV2* genes, which are known to be implicated in angiogenic-related processes (Lee et al., 2006; Linnemann et al., 2011).

In the case of mesenchymal cells, we identified >3.5-ES for three subclusters, such as RNA-Myogenesis (C4.1), Factors-Immune (C4.2), and ossification (D2). Importantly, some of the shared genes correspond to genes that allowed the subcluster labeling through GO categories (Figure 7B and Table S11). Genes such as *CXCL12, APOE,* or *IGFBP3* are associated with cell migration and lipid transport pathways among others (Amable et al., 2014; Robert et al., 2020), and characterized the murine adipogenesis subcluster (C2.3). Other genes, such as *IGFBP5*, are involved in actin filament assembly and organization (Sureshbabu et al., 2012) and defined the cell adhesion subcluster (C2.1). *IFIT3, MIDN, and ILR1* belong to pathways associated with interferon regulation or autoimmunity (Guo et al., 2017; Kim et al., 2020) and were identified in the immune response subclusters (C3 and C4.2). Additionally, the expression of *COL5A1* and *CADM1* genes, previously related to collagen fibril organization and bone mineralization processes (Kahai et al., 2004; Nakamura et al., 2017), defined the bone formation subcluster (D1). Moreover, genes such as *SPP1* or *CLEC11A*, which are related to osteoblasts function and mineralization (Huang et al., 2007; Shen et al., 2021; Yue et al., 2016), defined the mouse OLN-primed MSCs subcluster associated with ossification (D2) and are also expressed in human MSC-OLN cluster. Altogether, this data indicate the conservation of the osteogenic microenvironment between both species. To further explore the conservation of the BM microenvironment between both species, we examined the expression of known niche factors that regulate hematopoiesis. Despite the limited number of human cells, we were able to identify common niche-derived factors specific to the endothelium (SPARC, TGFB1, SELP, and EGFL7 among others) and the mesenchyme (including CXCL12, RARRES2, KITLG, and GAS6) (Figure 7C). These cytokines, secreted molecules and growth factors, represent key regulators of HSPCs shared among species.

Together, our analysis suggests that deep characterization of cellular states in mice can be used to infer conserved features in the human BM microenvironment despite a low level of conservation in the actual transcriptional profile of EC and higher in the case of MSC. Importantly, our data reveal a substantial degree of conservation regarding the complexity and heterogeneity of the EC and MSC compartment in the BM between mouse and human. This suggests that the layers of microenvironmental regulation of hematopoiesis and the identified plasticity in mice may also be shared between species.

## Discussion

Our study dissects the intrinsic organization and the heterogeneity within the endothelial (EC) and mesenchymal cell populations (MSC) governing the BM microenvironment. This was accomplished through customized bioinformatics integration of multiple datasets along with the inclusion of over 50.000 murine bone marrow stromal cells. We were able to identify new subsets of MSC and EC but, but more importantly, to define new molecular markers for the identification of highly specialized subpopulations of cells in the BM microenvironment. Pathway enrichment analysis unveiled multiple, potentially transient cell states defined by differential gene expression and the enrichment of specific functional characteristics. Importantly, EC subsets were characterized by enrichment in pathways known to be essential for endothelial homeostasis maintenance, demonstrating a high degree of specialization in the endothelium. Similarly, multiple transient cell states in the MSC compartment were defined and characterized by their differentiation capacity. Importantly, our deep deconvolution of the heterogeneous mesenchymal and endothelial compartments became feasible only by integrating multiple datasets. Of note, our analysis showcases that a research paradigm aiming for the generation of a detailed comprehensive molecular atlas of an organism requires both multi-omic data and computational integration. Here, we have relied on what is referred to as unpaired unimodal (scRNA-seq) data. Clearly, a natural next step is to use and further develop new computational tools that permit the integration (Argelaguet et al., 2021) of unpaired multi-omics datasets such as scRNA-seq, scATAC-seq, and other data modalities. Recent technological developments enable several multiple omics recorded from the same cell, i.e. paired data, which leverages our ability to dissect and molecularly characterize the intrinsic organization of the bone marrow niche environment. Advances in computational biology have started to develop such tools (Ashhurst et al., 2021; Hao et al., 2021; Martinez-de-Morentin et al., 2021; Wu et al., 2021). Moreover, some validation experiments, such as the use of fluorescent reporters, targeting niche-associated genes, and functional studies will allow us to confirm the identified new molecular markers based on differential gene expression and also the related annotated pathways.

While our study did not directly address the influence between stromal cells in the hematopoietic stem cell niche and the HSCs, the deep resolution of our study allows for some inferences to be made. Of note, we detected the expression of vascular endothelial growth factor-C (*Vegf-c*) in mouse endothelial and mesenchymal cells (Tables S2, S5, S6, S8, and S11). *Vegf-c* has recently been implicated in the maintenance of the perivascular niche and the recovery of hematopoiesis upon injury (Fang et al., 2020). *Vegf-c* is specifically expressed in the endothelial B1 and mesenchymal C1 subpopulations, suggesting an important role of these specialized endothelial and mesenchymal cells in the preservation of the integrity of the perivascular niche. In addition, Apelin+ (Apln) endothelial cells have been recently implicated in HSC maintenance and regeneration upon injury (Chen et al., 2019a). Importantly, two endothelial subclusters (B3.2-actin endocytosis and A1.1-wounding arteries) demonstrated expression of Apln, suggesting that these EC states represent specific sources of hematopoietic support and vascular regeneration upon injury. Osteolectin+ LepR–+mechanosensitive peri-arteriolar mesenchymal cells with osteogenic potential—are implicated in lymphoid, but not HSC maintenance (Shen et al., 2021). Importantly, osteolectin expression defines murine cluster D2 (ossification) and shows conservation in human MSCs, suggesting the preservation of a specialized lymphoid niche between species.

Detailed characterization of the human BM niche has not yet been addressed. Approaches undertaken in a mouse system cannot readily be transferred to the human system. Furthermore, differences in sample processing can also impact the results. In that sense, our results, despite the low number of cells, may represent the first dataset that includes scRNAseq from the human endothelial and mesenchymal BM microenvironment. While we were able to identify mesenchymal and endothelial cells based on canonical markers shared with mice (Aoki et al., 2021; Baryawno et al., 2019; Kalucka et al., 2020; Leimkühler et al., 2021; Matthews et al., 2020; Severe et al., 2019; Stumpf et al., 2020; Tikhonova et al., 2019; Wang et al., 2020; Xie et al., 2021), our human scRNAseq did not possess enough resolution to elucidate the heterogeneity of the human BM stroma to the same level as with the mouse data. Based on the extensive knowledge generated in the mouse, we therefore focused on characterizing how much of the information and targets from the mouse can be of interest in human characterization. This analysis allowed us to identify the expression of the human orthologs to the murine cluster-defining genes with different degrees of enrichment in the endothelium and mesenchyme. Some of these shared genes in mice and human stromal cells corresponded to the GO-defining genes of the different clusters identified in the mouse. Further, analysis of niche factors produced by ECs or MSCs and known to regulate hematopoiesis in mice were found conserved in the human samples. These findings suggest a meaningful degree of conservation regarding the cellular states that define the stromal microenvironment in mouse and human. Although additional studies and improved processing of human samples will be required for deep characterization of the human BM microenvironment, these preliminary results validate our integrative cross-species approach.

As an example of the added value, the current study identifies candidates of relevance in the study of BM-related diseases. *Sbds*, a ribosome maturation protein associated with the Shwachman-Diamond syndrome, represents a previously unrecognized marker of immature MSCs based on the dataset integration. Sbds deficiency has been implicated in ossification defects and metabolic changes in HSPCs (Raaijmakers et al., 2010; Zambetti et al., 2016), potentially contributing to myelodysplasia and AML onset in patients with Shwachman-Diamond syndrome. Future studies will help to improve the understanding of these new candidates and the pathogenesis associated. On a broader note, deep molecular analysis of the BM microenvironment sets the stage for computational disease modeling (Tegnér et al., 2009) from a systems medicine perspective.

Taken together, our study provides a deeper understanding of the composition and specialization of the BM microenvironment and point toward a substantial degree of conservation between species. Moreover, we demonstrate the usefulness of the multi-dataset integration and the customized clustering approach used in our study to improve the resolution of complex tissues and organs. This approach promises to aid in the construction of cell atlases by reducing the resources associated with sequencing that a single lab will need to invest in order to obtain meaningful depth in single-cell analysis while reducing the biases that may arise from data generated from a single laboratory or platform.

Future studies integrating genome, transcriptome, epigenome, proteome, and anatomical positioning together with functional assays to correlate descriptive phenotypes with functional data will help fully resolve the composition, regulation, and connectivity in the BM microenvironment in health and disease.

### Limitations of the study

This study provides the most comprehensive description to date of the murine HSC-regulatory microenvironment and suggests a higher level of specialization of the cellular circuits than previously anticipated. Moreover, this knowledge generated in the mouse allows the inference in human demonstrating substantial conservation between species. However, these studies are limited by the number of cells sequenced, the difficulty of obtaining high-quality samples with sufficient stromal cell numbers from healthy individuals, as well as the presence of contaminating populations in human data. The integrative analysis has limitations to account for the different.

**Abbreviations**

HSC Hematopoietic stem cell
HSPC Hematopoietic stem and progenitor cells
scRNA-seq Single-cell RNA sequencing
BM Bone marrow
EC Endothelial cells
MSC Mesenchymal cells
OLN-primed Osteolineage
MVG Most variable genes

## STAR★Methods

### Key resources table

**Table:** 

REAGENT or RESOURCE	SOURCE	IDENTIFIER
**Antibodies**		

Biotin anti-mouse lineage cocktail antibody (Mac1, CD3, Gr1, B220 and TER-119)	BIOLEGEND-DEDHAM	Cat#133307
APC/Cyanine7 Streptavidin	BIOLEGEND-DEDHAM	Cat#405208
Brilliant Violet 510 anti-mouse CD45 antibody (clone 30F11)	BIOLEGEND-DEDHAM	Cat#103137
APC anti-mouse CD45 antibody (clone 30F11)	BIOLEGEND-DEDHAM	Cat#103112
APC anti-mouse TER-119 antibody	BIOLEGEND-DEDHAM	Cat#116212
Brilliant Violet 510 anti-human CD3 antibody	BIOLEGEND-DEDHAM	Cat#317332
Brilliant Violet 510 anti-human CD19 antibody	BIOLEGEND-DEDHAM	Cat#363020
V500 anti-human CD45 antibody (Clone HI30)	BD horizon	Cat#560777
Brilliant Violet 510 anti-human CD64 antibody	BIOLEGEND-DEDHAM	Cat#305028
BV421 anti-human CD45 antibody	BIOLEGEND-DEDHAM	Cat#304032
BV421 anti-human CD235 antibody	BIOLEGEND-DEDHAM	Cat#349108
FITC anti-human CD31 antibody	BIOLEGEND-DEDHAM	Cat#303104
APC/FIRE 750 anti-human CD9 antibody	BIOLEGEND-DEDHAM	Cat#312113
PE 750 anti-human CD146 antibody	BIOLEGEND-DEDHAM	Cat#361005
PERCP Cy5.5 anti-human CD271 antibody	BIOLEGEND-DEDHAM	Cat#345112

**Biological samples**		

Bone marrow aspirates	healthy young donors	N/A

**Chemicals, peptides, and recombinant proteins**		

STREPTAVIDIN MICROBEADS	MILTENYI BIOTEC, S.L.	Cat#130-048-101
Vybrant DyeCycle Orange Stain	ThermoFisher Scientific	Cat#V35005
ANNEXIN V FITC	PHARMINGEN	Cat#556419
7AAD	BD Bioscience	Cat#559925
TO-PRO-3 IODIDE (642/661)	THERMO FISHER SCIENTIFIC S.L.	Cat#T3605

**Critical commercial assays**		

Miltenyi LD columns	MILTENYI BIOTEC, S.L.	Cat#130-042-901

**Deposited data**		

Raw and analyzed data	This paper	Single Cell Portal: SCP1747

**Experimental models: Organisms/strains**		

Female C57BL/6J mice, CD45.2	Jackson Laboratory	Cat#000664

**Software and algorithms**		

FlowJo (version 10.7.1) software	N/A	N/A
R (version 4.0.3, 3.6.3)	R core	https://www.r-project.org/
Seurat (version 4.0.0, 3.2.3)	Stuart et al. (2019)	https://github.com/satijalab/seurat/
SCTransform	Hafemeister and Satija (2019)	https://github.com/satijalab/sctransform
IKAP	Chen et al. (2019b)	https://github.com/GenomicsNX/IKAP
CellRanger (version 6.0.1)	10× Genomics	www.10xgenomics.com/
SingleR (version 1.4.1)	Aran et al. (2019)	https://github.com/dviraran/SingleR
ClusterProfiler (version 3.18.1)	Yu et al. (2012)	https://guangchuangyu.github.io/software/clusterProfiler/

### Resource availability

#### Lead contact

Further information and requests for resources and reagents should be directed to and will be fulfilled by the lead contact, Dr. David Gomez-Cabrero (david.gomezcabrero@kaust.edu.sa).

#### Materials availability

Requested materials are available from the lead contact.

### Experimental model and subject details

*For in vivo animal studies*, six females C57BL/6J mice (CD45.2) at age 20 weeks were used for Single-Cell RNA-seq experiments. Animals studies were carried out in accordance with the European Communities Council Directive (2010/63/UE) and with the approval of the Ethical Committee for Animal Testing of the University of Navarra. The number of mice per experiment was calculated to adhere to the “Replace, refine and reduce” rule for animal use in experimental procedures.

*For human studies*, a total volume of approximately 60 mL bone marrow (BM) was obtained by aspiration from the posterior iliac crest from four healthy young adults (20–30 years of age) after written informed consent was achieved. The human sample collection and research conducted in this study were approved by the Research Ethics Committee of the University of Navarra (project 2017.218). Personal data was kept confidential in accordance with the Organic law 3/2018, on personal data protection and Spanish law 14/2007 on Biomedical research. All collection samples are codified, and only the authorized personnel were able to correlate the patient’s identity with the codes.

### Method details

#### Isolation and FACS sorting of murine bone marrow microenvironment cells

Six mice were euthanized via CO_2_ asphyxiation. Bones from humerus, radius, iliac crests, femurs and tibia were harvested in PBS 1X containing 2% FBS and 2 mM EDTA (modPBS). All steps were performed on ice to preserve cell viability and RNA integrity. Muscles and soft tissue were thoroughly removed from the bones and bone marrow (BM) cells were obtained by crushing in modPBS. Cells were then filtered through a 70 μm cell strainer and red blood lysed with ACK buffer (NH_4_Cl 150 mM, KHCO_3_ 10 mM, and Na_2_EDTA 0.1 mM) for 10 min at room temperature (RT) with rotation. Remaining calcified bone fragments were collected on a 50 mL conical tube and digested with the appropriate volume of PBS with 0.3% collagenase I and dispase (5 U/mL) during 15 min at 37°C and shaking at 200 rpm. FBS representing 10% of the digestion volume was added to stop the collagenase digestion. After digestion, the calcified and crushed fractions were filtered through a 70 μm filter into a collection tube and pooled into one sample. Cells were subsequently stained for 20 min on ice first in the appropriate volume of modified PBS 1X (3 mL/mouse) with 160 ul/mouse of biotinylated lineage cocktail (Mac1, CD3, Gr1, B220 and TER119) followed by incubation with streptavidin magnetics microbeads (100 μL/mouse). Negative selection was performed using Miltenyi LD columns according to manufacturers’ protocol. After selection, the sample was stained with the following combination of conjugated antibodies at a concentration of 1/200: APC-Cy7 labeled streptavidin, BV510 labeled anti-CD45, APC labeled anti-CD45 and APC labeled anti-TER-119. Samples were then stained with 0.05 μM of Vybrant dye orange (VDO) at 37°C for 30 min to label living cells. Annexin V was also added, in combination with 7AAD to discard apoptotic and dead cells from the sample respectively. For annexin V staining, cells were stained with 1 μL/mouse of Annexin V-FITC on an appropriate volume of 1X Annexin V binding buffer in the dark for 15 min at RT. Samples were then resuspended in 1X Annexin V buffer and 5 μL of 7AAD dye (up to 1 × 10^6^ cells) were added. BM non-hematopoietic cells were FACS sorted using BD FACSAria II sorter collected in PBS 1X supplemented with 0.05% UltraPure BSA and cell viability was assessed using Nexcelom Cellometer. Data were analyzed by FlowJo (version 10.7.1) software.

#### Isolation and FACS sorting of human bone marrow endothelial and mesenchymal-osteolineage cells

All sample processing steps were performed on ice to preserve cell viability and RNA integrity. A total volume of approximately 60 mL of bone marrow was obtained by aspiration from the posterior iliac crest. Red blood cells were lysed twice with 45 mL of ACK buffer per 5 mL of human sample during 15 min at RT with rotation. Sample was then filtered through a 70 μm cell strainer, centrifuged, and stained for 30 min on ice with the following combination of conjugated antibodies at a concentration of 1/100 except anti-Lin (3ul/test- test 25 × 10^6^cells): BV510 labeled anti-Lin (including CD3, CD10, CD19, CD45 and CD64), BV421 labeled anti-CD235, BV421 labeled anti- CD45, FITC labeled anti-CD31, APC-Cy7 labeled anti-CD9, PE labeled anti-CD146 and PerCP- Cy5.5 labeled anti-CD271. Dead cells and debris were firstly excluded by FSC, SSC and adding 10 μL of TO-PRO-3. BM niche populations were prospectively isolated based on the following immunophenotype: ECs: TO-PRO-3^-^/Lin^−^/CD45^-^/CD235^-^/CD9^+^/CD31^+^ and MSCs: TO-PRO-3^-^/Lin^−^/CD45^-^/CD235^-^/CD31^-^/CD271^+^/CD146^+/−^. FACS sorting was performed on a BD FACSAria II sorter, sorted BM niche cells were collected in PBS 1× and 0.05% Ultra-Pure BSA and cell viability was determined using Nexcelom Cellometer. Data were analyzed by FlowJo (version 10.7.1) software.

#### Profiling by single-cell RNA-sequencing (scRNA-seq)

scRNA-seq was performed using the Single Cell 3′ Reagent Kits v3.1 (10X Genomics) according to the manufacturer’s instructions. For human samples, endothelial and mesenchymal cells were pooled before scRNA-seq was performed. Approximately 15,000 cells were loaded at a concentration of 1,000 cells/μL on a Chromium Controller instrument (10X Genomics) to generate single-cell gel bead-in-emulsions (GEMs). In this step, each cell was encapsulated with primers containing a fixed Illumina Read 1 sequence, followed by a cell-identifying 16 bp 10X barcode, a 10 bp Unique Molecular Identifier (UMI) and a poly-dT sequence. A subsequent reverse transcription yielded full-length, barcoded cDNA. This cDNA was then released from the GEMs, PCR-amplified and purified with magnetic beads (SPRIselect, Beckman Coulter). Enzymatic Fragmentation and Size Selection was used to optimize cDNA size prior to library construction. Illumina adaptor sequences were added, and the resulting library was amplified via end repair, A- tailing, adaptor ligation and PCR. Libraries quality control and quantification was performed using Qubit 3.0 Fluorometer (Life Technologies) and Agilent’s 4200 TapeStation System (Agilent), respectively. Sequencing was performed in a NextSeq500 (Illumina) (Read 1: 26 cycles, i7 Index: 8 cycles, Read 2: 49 cycles) at an average depth of 60,000 reads/cell in mice and 30,000 reads/cell in human.

#### Single-cell RNA-seq analysis of mouse samples

See extended details and code in the following Github: https://github.com/TranslationalBioinformaticsUnit/BMN_characterization.

##### Sample selection

Sample GSM3674224, GSM3674225, GSM3674226, GSM3674227, GSM3674228, GSM3674229 from GSE128423 by Baryawno, sample GSM2915575, GSM2915576, GSM2915577 from GSE108891 by Tikhonova and one in-house mouse bone marrow niche sample was included in this analysis.

##### Filtering

The single cell analysis of mice samples analysis was performed using R (version 4.0.3 for human, 3.6.3 for mouse) and Seurat (version 4.0.0 for human, 3.2.3 for mouse)(Stuart et al., 2019).Three bone marrow niche samples were filtered individually based on the 10^th^ and 90^th^ quantile of number of features and counts. Cells with more than 5% mitochondrial genes were also removed. Each dataset was normalized using SCTransform function (Hafemeister and Satija, 2019) from Seurat package separately.

##### Pairwise integration and selection of the target population

In-house dataset and Baryawno were integrated with Tikhonova separately using IntegrateData function from Seurat (version 3.2.3.). Using as a reference the annotation from Tikhonova dataset, cells that aligned with LEPR+ cells and VE-Cad+ cells were annotated as MSC and EC respectively. MSC-like cells and EC-like cells from different datasets were normalized again and integrated using Seurat without further filtering.

##### Clustering

After filtering and quality control, a divide-and-conquer strategy was applied to the clustering of mouse ECs and MSCs separately. Firstly, following integration, dimension reduction with principal component analysis (PCA), data visualization with Uniform Manifold Approximation and Projection (UMAP), computation of K-nearest neighbors and an initial Louvain clustering using resolution of 1 were performed as a reference of high-resolution limit. Secondly, IKAP (Chen et al., 2019b) was applied to each integrated dataset as “level 1” clustering (Figure 2B). Each cluster from level 1 was then compare with the high-resolution reference. The cluster from level 1 was further divided using IKAP to level 2 if the cluster were far from cluster limit. The process would be repeated until at least one cluster reach cluster limit.

##### Cluster evaluation

To evaluate the stability of these clusters, a bootstrapping strategy was adopted (Tasic et al., 2016). The script was rewritten for our pipeline, and we further defined two metrics: (a) recall per cell and (b) #Correct (details below) for each cluster to obtain better demonstration and quantification of the cluster robustness.

The strategy was conducted in a pairwise manner with basic steps as follow:

1. Select two clusters, randomly split the clusters to five equal groups and use one group of cells (20%) as testing dataset.
2. Identify the set of differentially expressed genes (DEGs) between the 2 clusters using the Wilcoxon Rank Sum test (Wilcoxon, 1945).
3. Train a random forest classifier with 20 genes selecting the top 10 DEGs from each cluster based on average log2 fold change.
4. Applied the classifier to the 20% testing dataset.
5. Repeat step1-4 for five times for different groups such that each cell in these two clusters was classified once.
6. Repeat step1-5 nine more times.
7. Repeat step1-6 for all cluster pairs

There are three types of results that can be summarized from this bootstrapping strategy:

1) Dominant cluster identification: A dominant cluster is the cluster to which the cell is assigned for more than half the runs.
2) Number of Correct Dominance Assignment (#Correct): The sum of times the dominant cluster matches the original cluster (true positive) for a cell across all cluster pair comparisons (Figures S2E and S2H).
3) Recall Per Cell: The proportion of correct assignment (positive result) to its original cluster in all runs from all comparisons (Figures S2D and S2G).

Clusters where more than 50% of the cells has been “incorrectly” assigned robustly at least once to another dominant cluster will be considered unstable (Figures S2F and S2H, cluster A2.2). The cells of such cluster will be assigned to other clusters (see dominant plots, Figure S9 as an example) using – to that end - a random forest classifier as described before.

To evaluate the mixing of three dataset in each cluster after the integration, Shannon entropy was calculated. For each cell j in a cluster, the probability of them coming from any of the datasets (d = 1,2,3) is denoted as $p_{j}^{d}$, the entropy for this cell was calculated as $H_{j}=-\overset{3}{\underset{d=1}{\sum }}p_{j}^{d}logp_{j}^{d}$.

##### Gene set analysis

After clustering, DEGs of each cluster were identified using a Wilcoxon Rank Sum test. For each cluster, an over-representation analysis of GO gene set enrichment analysis was conducted using the top 30, 50 and 100 DEGs based on the average log transformed fold changes using clusterProfiler (version 3.18.1)(Yu et al., 2012). Additionally, to further unveil the specialization of those subclusters, gene sets were also computed within the sub-divisions of each cluster at the last clustering level with the top 30, 50, 100 DEGs. Final annotation of each cluster was manually assigned based on conserved gene terms within all the analysis.

#### Added value analysis

##### Added value 1

Comparing the DEGs defined by individual dataset and integrated dataset. The individual datasets were normalized with SCTransform and DEGs were identified within top 3000 most variable genes using a Wilcoxon Rank Sum test. For integrated dataset, DEGs were identified within top 3000 most variable genes from integrated assay using a Wilcoxon Rank Sum test. False negative and false positive rate were calculated by comparing the DEGs identified by integrated dataset and individual dataset (Figures 3E and 3F).

##### Added value 2

Cluster stability evaluation for individual dataset. To understand if the clusters identified from three datasets can remain stable within a single dataset or not, the bootstrapping strategy was applied to each dataset with the annotation identified by the integrated dataset.

##### Added value 3

Comparing cluster identified by a single dataset. To further understand if the clusters can be identified by one dataset only, Baryawno dataset was used as an example considering its large cell populations. The same pipeline from normalization to bootstrapping was applied to this dataset and the clusters identified from this single dataset was compared with the clusters identified by three datasets using Jaccard index.

##### Added value 4

Application of cluster identity in a separate dataset (Baccin et al., 2019). The clusters identified were used as reference to annotate ECs and MSCs from Baccin data using singleR (version 1.4.1)(Aran et al., 2019).Normalized counts in “RNA” assay from Seurat object were used for this analysis.

#### Single-cell RNA-seq analysis of human samples

Preprocessing of sequencing data: Preprocessing of single-cell RNA-seq data for each in house sample were conducted by CellRanger count from Cell Ranger (version 6.0.1) using reference genome GRCh38.

##### Sample filtering

The single cell analysis of human analysis was performed as described in before except for human cells with more than 10% mitochondrial genes were also removed. Because the exploratory data analysis revealed potential contamination of B cells, we applied an additional filter: cells with more than 10% reads mapped to immunoglobin genes were excluded from downstream analysis.

##### Integration

After filtering, each sample was normalized using SCTransform and integrated using 3000 most variable features using Seurat. Following integration, dimension reduction with PCA, data visualization with UMAP, computation of K-nearest neighbors with 20 dimensions and clustering using resolution of 0.4 were performed.

##### Select EC and MSC (without further integration)

Clusters were annotated based on biological insights on markers. Cluster 11 were identified as *mesenchymal* and cluster 1, 6 were identified as *endothelial*. During the exploratory analysis, human EC cells were subclustered at resolution 0.4 and one of the clusters identified was further filtered for the downstream analysis because the cells in the cluster were not expressing EC marker genes. Several outliers from human MSC cells were also removed.

##### Compare human MVGs with mouse DEGs

3000 MVGs for human EC and MSC were identified using “RNA” assay and these genes were scaled in “integrated” assay resulting in 932 human EC specific MVGs and 976 human MSC specific MVGs. The MVGs from human EC or MSC were compared with DEGs from each mouse cluster. The enrichment score for a given cluster *i* was defined as the ratio between “*the number of genes shared between human MVGs and mouse DEGs from cluster i*” and “*the expected number of genes*”, where the later was computed as follows:

$$\frac{MVG_{\mathrm{human}}\cap MVG_{\mathrm{mouse}}\;}{MVG_{\mathrm{mouse}}}\times \;l_{i}$$

Where $l_{i}$ is the number of DEGs from mouse cluster i.

#### SingleR analysis between mouse and human

To annotate human cells using mouse clusters as reference the singleR tool (version 1.4.1)(Aran et al., 2019) was utilized for ECs and MSCs separately. Cell type specific MVGs with expression values in “integrated” assay from Seurat object were used for this analysis.

### Quantification and statistic analysis

Statistical analysis in this study (t-test and comparing proportions) were conducted using R.

#### Ethics approval

All animal experiments were performed in accordance with national and institutional guidelines and procedures were approved by the Ethical Committee for Animal Testing of the University of Navarra. Acknowledging the principles of 3Rs (Replacement, Reduction and Refinement), all mice used in this study were from mice that were euthanized by cervical dislocation as parts of other ongoing ethically approved experiments.

The human sample collection and research conducted in this study were approved by the Research Ethics Committee of the University of Navarra. All the protocols used in this study were in strict compliance with the legal and ethical regulations.

## Data Availability

• Public data was gathered from the following NCBI GEO accessing numbers: samples GSM3674224, GSM3674225, GSM3674226, GSM3674227, GSM3674228, and GSM3674229 from GSE12842; samples GSM2915575, GSM2915576, and GSM2915577 from GSE108891. Additionally, we profiled an in-house mouse (one sample) and human (4 samples) bone marrow niche sample; Raw count table of human and in-house mouse samples in this study are available in Single Cell Portal with accession number SCP1747 (https://singlecell.broadinstitute.org/single_cell/study/SCP1747/bmn-characterization). Any additional information required to reanalyze the data reported in this work paper is available from the lead contact upon request. • The code used in this analysis is available at: https://github.com/TranslationalBioinformaticsUnit/BMN_characterization.

## Supplemental information

Document S1. Figures S1–S9

Table S9. Gene Set Enrichment in Human integrated data (GSE Human), related to Figure 6

Table S10. Genes from mouse endothelial clusters in Human data (mEC genes Human), related to Figure 7

Table S11. Genes from mouse mesenchymal clusters in Human data (mMSC genes Human), related to Figure 7

Table S1. Entropy measured by subcluster, related to Figure 3

Table S2. Marker genes per cluster in Mouse endothelial cells (EC markers), related to Figure 4

Table S3. Marker genes between sinusoids and arteries in Mouse endothelial cells (Sin vs Art), related to Figure 4

Table S4. Gene-Set Enrichment per cluster in Mouse endothelial cells (GSE EC), related to Figure 4

Table S5. Marker genes per cluster in Mouse Mesenchymal cells (MSC markers), related to Figure 5

Table S6. Marker genes between mesenchymal and osteolineage in Mouse Mesenchymal cells (MSC vs OLN), related to Figure 5

Table S7. Gene-Set Enrichment per cluster in Mouse Mesenchymal cells (GSE MSC), related to Figure 5

Table S8. Human Cluster Marker after IG Filtering (Human Cluster Marker), related to Figure 6

## References

[bib1] Amable P.R., Teixeira M.V.T., Carias R.B.V., Granjeiro J.M., Borojevic R. (2014). Gene expression and protein secretion during human mesenchymal cell differentiation into adipogenic cells. BMC Cell Biol..

[bib2] Aoki K., Kurashige M., Ichii M., Higaki K., Sugiyama T., Kaito T., Ando W., Sugano N., Sakai T., Shibayama H. (2021). Identification of CXCL12-abundant reticular cells in human adult bone marrow. Br. J. Haematol..

[bib3] Aran D., Looney A.P., Liu L., Wu E., Fong V., Hsu A., Chak S., Naikawadi R.P., Wolters P.J., Abate A.R. (2019). Reference-based analysis of lung single-cell sequencing reveals a transitional profibrotic macrophage. Nat. Immunol..

[bib4] Argelaguet R., Cuomo A.S.E., Stegle O., Marioni J.C. (2021). Computational principles and challenges in single-cell data integration. Nat. Biotechnol..

[bib5] Ashhurst T.M., Marsh-Wakefield F., Putri G.H., Spiteri A.G., Shinko D., Read M.N., Smith A.L., King N.J.C. (2021). Integration, exploration, and analysis of high-dimensional single-cell cytometry data using Spectre. Cytometry A.

[bib6] Baccin C., Al-Sabah J., Velten L., Helbling P.M., Grünschläger F., Hernández-Malmierca P., Nombela-Arrieta C., Steinmetz L.M., Trumpp A., Haas S. (2020). Combined single-cell and spatial transcriptomics reveal the molecular, cellular and spatial bone marrow niche organization. Nat. Cell Biol..

[bib7] Baccin C., Al-Sabah J., Velten L., Helbling P.M., Grünschläger F., Hernández-Malmierca P., Nombela-Arrieta C., Steinmetz L.M., Trumpp A., Haas S. (2019). Combined single-cell and spatial transcriptomics reveal the molecular, cellular and spatial bone marrow niche organization. Nat. Cell Biol..

[bib8] Barreiro O., Yáñez-Mó M., Sala-Valdés M., Gutiérrez-López M.D., Ovalle S., Higginbottom A., Monk P.N., Cabañas C., Sánchez-Madrid F. (2005). Endothelial tetraspanin microdomains regulate leukocyte firm adhesion during extravasation. Blood.

[bib9] Barrientos T., Frank D., Kuwahara K., Bezprozvannaya S., Pipes G.C.T., Bassel-Duby R., Richardson J.A., Katus H.A., Olson E.N., Frey N. (2007). Two novel members of the ABLIM protein family, ABLIM-2 and -3, associate with STARS and directly bind F-actin. J. Biol. Chem..

[bib10] Baryawno N., Przybylski D., Kowalczyk M.S., Kfoury Y., Severe N., Gustafsson K., Kokkaliaris K.D., Mercier F., Tabaka M., Hofree M. (2019). A cellular taxonomy of the bone marrow stroma in homeostasis and leukemia. Cell.

[bib11] Blondel V.D., Guillaume J.-L., Lambiotte R., Lefebvre E. (2008). Fast unfolding of communities in large networks. J. Stat. Mech. Theor. Exp..

[bib12] Chen Q., Liu Y., Jeong H.W., Stehling M., Dinh V.V., Zhou B., Adams R.H. (2019). Apelin+ endothelial niche cells control hematopoiesis and mediate vascular regeneration after myeloablative injury. Cell Stem Cell.

[bib13] Chen Y.C., Suresh A., Underbayev C., Sun C., Singh K., Seifuddin F., Wiestner A., Pirooznia M. (2019). IKAP-Identifying K mAjor cell Population groups in single-cell RNA-sequencing analysis. GigaScience.

[bib14] Davis G.E., Senger D.R. (2005). Endothelial extracellular matrix: biosynthesis, remodeling, and functions during vascular morphogenesis and neovessel stabilization. Circ. Res..

[bib15] Dolgalev I., Tikhonova A.N. (2021). Connecting the dots: resolving the bone marrow niche heterogeneity. Front. Cell Develop. Biol..

[bib16] Echavarria R., Hussain S.N.A. (2013). Regulation of angiopoietin-1/Tie-2 receptor signaling in endothelial cells by dual-specificity phosphatases 1, 4, and 5. J. Am. Heart Assoc..

[bib17] Escudero-Esparza A., Jiang W.G., Martin T.A. (2012). Claudin-5 participates in the regulation of endothelial cell motility. Mol. Cell Biochem..

[bib18] Fang S., Chen S., Nurmi H., Leppänen V.M., Jeltsch M., Scadden D., Silberstein L., Mikkola H., Alitalo K. (2020). VEGF-C protects the integrity of the bone marrow perivascular niche in mice. Blood.

[bib19] Ghazanfari R., Li H., Zacharaki D., Lim H.C., Scheding S. (2016). Human non-hematopoietic CD271pos/CD140alow/neg bone marrow stroma cells fulfill stringent stem cell criteria in serial transplantations. Stem Cells Develop..

[bib20] Giladi A., Paul F., Herzog Y., Lubling Y., Weiner A., Yofe I., Jaitin D., Cabezas-Wallscheid N., Dress R., Ginhoux F. (2018). Single-cell characterization of haematopoietic progenitors and their trajectories in homeostasis and perturbed haematopoiesis. Nat. Cell Biol..

[bib21] Gomez-Cabrero D., Menche J., Cano I., Abugessaisa I., Huertas-Migueláñ M., Tenyi A., de Mas I.M., Kiani N.A., Marabita F., Falciani F. (2014). Systems Medicine: from molecular features and models to the clinic in COPD. J. Transl. Med..

[bib22] Guo W., Imai S., Yang J.L., Zou S., Watanabe M., Chu Y.X., Mohammad Z., Xu H., Moudgil K.D., Wei F. (2017). In vivo immune interactions of multipotent stromal cells underlie their long-lasting pain-relieving effect. Sci. Rep..

[bib23] Hafemeister C., Satija R. (2019). Normalization and variance stabilization of single-cell RNA-seq data using regularized negative binomial regression. Genome Biol..

[bib24] Hajjawi M.O.R., MacRae V.E., Huesa C., Boyde A., Millán J.L., Arnett T.R., Orriss I.R. (2014). Mineralisation of collagen rich soft tissues and osteocyte lacunae in Enpp1-/- mice. Bone.

[bib25] Hamidouche Z., Fromigué O., Ringe J., Häupl T., Vaudin P., Pagès J.C., Srouji S., Livne E., Marie P.J. (2009). Priming integrin α5 promotes human mesenchymal stromal cell osteoblast differentiation and osteogenesis. Proc. Natl. Acad. Sci. U S A.

[bib26] Hao Y., Hao S., Andersen-Nissen E., Mauck W.M., Zheng S., Butler A., Lee M.J., Wilk A.J., Darby C., Zager M. (2021). Integrated analysis of multimodal single-cell data. Cell.

[bib27] Hashemi S., Trudel E., Ganz P.R., Drouin J., Couture R., Page D., Aye M.T. (1991). Characterization of novel platelet and endothelial cell target antigens in a family with genetic susceptibility to autoimmunity. Am. J. Hematol..

[bib28] Hooper A.T., Butler J.M., Nolan D.J., Kranz A., Iida K., Kobayashi M., Kopp H.G., Shido K., Petit I., Yanger K. (2009). Engraftment and reconstitution of hematopoiesis is dependent on VEGFR2-mediated regeneration of sinusoidal endothelial cells. Cell Stem Cell.

[bib29] Huang W., Yang S., Shao J., Li Y.P. (2007). Signaling and transcriptional regulation in osteoblast commitment and differentiation. Front. Biosci..

[bib30] Itkin T., Gur-Cohen S., Spencer J.A., Schajnovitz A., Ramasamy S.K., Kusumbe A.P., Ledergor G., Jung Y., Milo I., Poulos M.G. (2016). Distinct bone marrow blood vessels differentially regulate haematopoiesis. Nature.

[bib31] Jia J., Ye T., Cui P., Hua Q., Zeng H., Zhao D. (2016). AP-1 transcription factor mediates VEGF-induced endothelial cell migration and proliferation. Microvasc. Res..

[bib32] Kahai S., Vary C.P.H., Gao Y., Seth A. (2004). Collagen, type V, α1 (COL5A1) is regulated by TGF-β in osteoblasts. Matrix Biol..

[bib33] Kalucka J., de Rooij L.P.M.H., Goveia J., Rohlenova K., Dumas S.J., Meta E., Conchinha N.V., Taverna F., Teuwen L.A., Veys K. (2020). Single-cell transcriptome atlas of murine endothelial cells. Cell.

[bib34] Kanazawa S., Okada H., Hojo H., Ohba S., Iwata J., Komura M., Hikita A., Hoshi K. (2021). Mesenchymal stromal cells in the bone marrow niche consist of multi-populations with distinct transcriptional and epigenetic properties. Sci. Rep..

[bib35] Karamitros D., Stoilova B., Aboukhalil Z., Hamey F., Reinisch A., Samitsch M., Quek L., Otto G., Repapi E., Doondeea J. (2017). Single-cell analysis reveals the continuum of human lympho-myeloid progenitor cells. Nat. Immunol..

[bib36] Kenswil K.J.G., Jaramillo A.C., Ping Z., Chen S., Hoogenboezem R.M., Mylona M.A., Adisty M.N., Bindels E.M.J., Bos P.K., Stoop H. (2018). Characterization of endothelial cells associated with hematopoietic niche formation in humans identifies IL-33 as an anabolic factor. Cell Rep..

[bib37] Kim S.H., Choi H., Choi M.R., An G.Y., Binas B., Jung K.H., Chai Y.G. (2020). Epigenetic regulation of IFITM1 expression in lipopolysaccharide-stimulated human mesenchymal stromal cells. Stem Cell Res. Ther..

[bib38] Klóska D., Kopacz A., Piechota-Polańczyk A., Neumayer C., Huk I., Dulak J., Józkowicz A., Grochot-Przęczek A. (2019). Biliverdin reductase deficiency triggers an endothelial-to-mesenchymal transition in human endothelial cells. Arch. Biochem. Biophys..

[bib39] Laurenti E., Göttgens B. (2018). From haematopoietic stem cells to complex differentiation landscapes. Nature.

[bib40] Lee J.S., Yu Q., Shin J.T., Sebzda E., Bertozzi C., Chen M., Mericko P., Stadtfeld M., Zhou D., Cheng L. (2006). Klf2 is an essential regulator of vascular hemodynamic forces *in vivo*. Develop. Cell.

[bib41] Leimkühler N.B., Gleitz H.F.E., Ronghui L., Snoeren I.A.M., Fuchs S.N.R., Nagai J.S., Banjanin B., Lam K.H., Vogl T., Kuppe C. (2021). Heterogeneous bone-marrow stromal progenitors drive myelofibrosis via a druggable alarmin axis. Cell Stem Cell.

[bib42] Linnemann A.K., O’Geen H., Keles S., Farnham P.J., Bresnick E.H. (2011). Genetic framework for GATA factor function in vascular biology. Proc. Natl. Acad. Sci. U S A.

[bib43] Mackenzie N.C.W., Zhu D., Milne E.M., van’t Hof R., Martin A., Quarles D.L., Millán J.L., Farquharson C., MacRae V.E. (2012). Altered bone development and an increase in FGF-23 expression in Enpp1 -/- mice. PLoS One.

[bib44] Macosko E.Z., Basu A., Satija R., Nemesh J., Shekhar K., Goldman M., Tirosh I., Bialas A.R., Kamitaki N., Martersteck E.M. (2015). Highly parallel genome-wide expression profiling of individual cells using nanoliter droplets. Cell.

[bib45] March S., Hui E.E., Underhill G.H., Khetani S., Bhatia S.N. (2009). Microenvironmental regulation of the sinusoidal endothelial cell phenotype *in vitro*. Hepatology.

[bib46] Martinez-de-Morentin X., Khan S.A., Lehmann R., Tegner J., Gomez-Cabrero D. (2021). Machine translation between paired single cell multi omics data. bioRxiv.

[bib47] Matsushita Y., Nagata M., Kozloff K.M., Welch J.D., Mizuhashi K., Tokavanich N., Hallett S.A., Link D.C., Nagasawa T., Ono W., Ono N. (2020). A Wnt-mediated transformation of the bone marrow stromal cell identity orchestrates skeletal regeneration. Nat. Commun..

[bib48] Matthews E., Lanham S., White K., Kyriazi M.E., Alexaki K., El-Sagheer A.H., Brown T., Kanaras A.G., West J., MacArthur B.D. (2020). Single cell RNA sequence analysis of human bone marrow samples reveals new targets for isolation of skeletal stem cells using DNA-coated gold nanoparticles. bioRxiv.

[bib49] Naito H., Iba T., Takakura N. (2021). Mechanisms of new blood-vessel formation and proliferative heterogeneity of endothelial cells. Int. Immunol..

[bib50] Nakamura S., Koyama T., Izawa N., Nomura S., Fujita T., Omata Y., Minami T., Matsumoto M., Nakamura M., Fujita-Jimbo E. (2017). Negative feedback loop of bone resorption by NFATc1-dependent induction of Cadm1. PLoS One.

[bib51] Nestorowa S., Hamey F.K., Pijuan Sala B., Diamanti E., Shepherd M., Laurenti E., Wilson N.K., Kent D.G., Göttgens B. (2016). A single-cell resolution map of mouse hematopoietic stem and progenitor cell differentiation. Blood.

[bib52] Nilius B., Droogmans G. (2001). Ion channels and their functional role in vascular endothelium. Physiol. Rev..

[bib53] Okamoto T., Suzuki K. (2017). The role of gap junction-mediated endothelial cell-cell interaction in the crosstalk between inflammation and blood coagulation. Int. J. Mol. Sci..

[bib54] Price P.A. (1985). Vitamin K-dependent formation of bone Gla protein (osteocalcin) and its function. Vitamins Horm..

[bib55] Quillon A., Fromy B., Debret R. (2015). Endothelium microenvironment sensing leading to nitric oxide mediated vasodilation: a review of nervous and biomechanical signals. Nitric Oxide Biol. Chem..

[bib56] Quirici N., Soligo D., Bossolasco P., Servida F., Lumini C., Deliliers G.L. (2002). Isolation of bone marrow mesenchymal stem cells by anti-nerve growth factor receptor antibodies. Exp. Hematol..

[bib57] Raaijmakers M.H.G.P., Mukherjee S., Guo S., Zhang S., Kobayashi T., Schoonmaker J.A., Ebert B.L., Al-Shahrour F., Hasserjian R.P., Scadden E.O. (2010). Bone progenitor dysfunction induces myelodysplasia and secondary leukaemia. Nature.

[bib58] Rafii S., Butler J.M., Sen D.B. (2016). Angiocrine functions of organ-specific endothelial cells. Nature.

[bib59] Robert A.W., Marcon B.H., Dallagiovanna B., Shigunov P. (2020). Adipogenesis, osteogenesis, and chondrogenesis of human mesenchymal stem/stromal cells: a comparative transcriptome approach. Front. Cell Develop. Biol..

[bib60] Rodriguez-Fraticelli A.E., Wolock S.L., Weinreb C.S., Panero R., Patel S.H., Jankovic M., Sun J., Calogero R.A., Klein A.M., Camargo F.D. (2018). Clonal analysis of lineage fate in native haematopoiesis. Nature.

[bib61] Severe N., Karabacak N.M., Gustafsson K., Baryawno N., Courties G., Kfoury Y., Kokkaliaris K.D., Rhee C., Lee D., Scadden E.W. (2019). Stress-Induced changes in bone marrow stromal cell populations revealed through single-cell protein expression mapping. Cell Stem Cell.

[bib62] Shen B., Tasdogan A., Ubellacker J.M., Zhang J., Nosyreva E.D., Du L., Murphy M.M., Hu S., Yi Y., Kara N. (2021). A mechanosensitive peri-arteriolar niche for osteogenesis and lymphopoiesis. Nature.

[bib63] Spohn G., Kleinridders A., Wunderlich F.T., Watzka M., Zaucke F., Blumbach K., Geisen C., Seifried E., Müller C., Paulsson M. (2009). VKORC1 deficiency in mice causes early postnatal lethality due to severe bleeding. Thromb. Haemost..

[bib64] Stuart T., Butler A., Hoffman P., Hafemeister C., Papalexi E., Mauck W.M., Hao Y., Stoeckius M., Smibert P., Satija R. (2019). Comprehensive integration of single-cell data. Cell.

[bib65] Stumpf P.S., Du X., Imanishi H., Kunisaki Y., Semba Y., Noble T., Smith R.C.G., Rose-Zerili M., West J.J., Oreffo R.O.C. (2020). Transfer learning efficiently maps bone marrow cell types from mouse to human using single-cell RNA sequencing. Commun. Biol..

[bib66] Sureshbabu A., Okajima H., Yamanaka D., Tonner E., Shastri S., Maycock J., Szymanowska M., Shand J., Takahashi S.I., Beattie J. (2012). IGFBP5 induces cell adhesion, increases cell survival and inhibits cell migration in MCF-7 human breast cancer cells. J. Cell Sci..

[bib67] Tamura K., Hashimoto K., Suzuki K., Yoshie M., Kutsukake M., Sakurai T. (2009). Insulin-like growth factor binding protein-7 (IGFBP7) blocks vascular endothelial cell growth factor (VEGF)-induced angiogenesis in human vascular endothelial cells. Eur. J. Pharmacol..

[bib68] Tasic B., Menon V., Nguyen T.N., Kim T.K., Jarsky T., Yao Z., Levi B., Gray L.T., Sorensen S.A., Dolbeare T. (2016). Adult mouse cortical cell taxonomy revealed by single cell transcriptomics. Nat. Neurosci..

[bib69] Tegnér J.N., Compte A., Auffray C., An G., Cedersund G., Clermont G., Gutkin B., Oltvai Z.N., Stephan K.E., Thomas R., Villoslada P. (2009). Computational disease modeling – fact or fiction?. BMC Syst. Biol..

[bib70] Testa U., Labbaye C., Castelli G., Pelosi E. (2016). Oxidative stress and hypoxia in normal and leukemic stem cells. Exp. Hematol..

[bib71] Tikhonova A.N., Dolgalev I., Hu H., Sivaraj K.K., Hoxha E., Cuesta-Domínguez Á., Pinho S., Akhmetzyanova I., Gao J., Witkowski M. (2019). The bone marrow microenvironment at single-cell resolution. Nature.

[bib72] Traag V.A., Waltman L., Eck NJ van (2019). From Louvain to Leiden: guaranteeing well-connected communities. Sci. Rep..

[bib73] Velten L., Haas S.F., Raffel S., Blaszkiewicz S., Islam S., Hennig B.P., Hirche C., Lutz C., Buss E.C., Nowak D. (2017). Human haematopoietic stem cell lineage commitment is a continuous process. Nat. Cell Biol..

[bib74] Wang Z., Li X., Yang J., Gong Y., Zhang H., Qiu X., Liu Y., Zhou C., Chen Y., Greenbaum J. (2020). Single-cell RNA sequencing deconvolutes the *in vivo* heterogeneity of human bone marrow-derived mesenchymal stem cells. bioRxiv.

[bib75] Weinreb C., Rodriguez-Fraticelli A., Camargo F.D., Klein A.M. (2020). Lineage tracing on transcriptional landscapes links state to fate during differentiation. Science.

[bib76] Wilcoxon F. (1945). Individual comparisons by ranking methods. Biometrics Bull..

[bib77] Wolkenhauer O., Auffray C., Jaster R., Steinhoff G., Dammann O. (2013). The road from systems biology to systems medicine. Pediatr. Res..

[bib78] Wolock S.L., Krishnan I., Tenen D.E., Matkins V., Camacho V., Patel S., Agarwal P., Bhatia R., Tenen D.G., Klein A.M., Welner R.S. (2019). Mapping distinct bone marrow niche populations and their differentiation paths. Cell Rep..

[bib79] Wu K.E., Yost K.E., Chang H.Y., Zou J. (2021). BABEL enables cross-modality translation between multiomic profiles at single-cell resolution. Proc Natl Acad Sci U S A.

[bib80] Xie J., Lou Q., Zeng Y., Liang Y., Xie S., Xu Q., Yuan L., Wang J., Jiang L., Mou L. (2021). Single-cell atlas reveals fatty acid metabolites regulate the functional heterogeneity of mesenchymal stem cells. Front. Cell Develop. Biol..

[bib81] Yau J.W., Teoh H., Verma S. (2015). Endothelial cell control of thrombosis. BMC Cardiovasc. Disord..

[bib82] Yu G., Wang L.G., Han Y., He Q.Y. (2012). ClusterProfiler: an R package for comparing biological themes among gene clusters. OMICS A J. Integr. Biol..

[bib83] Yue R., Shen B., Morrison S.J. (2016). Clec11a/osteolectin is an osteogenic growth factor that promotes the maintenance of the adult skeleton. ELife.

[bib84] Zambetti N.A., Ping Z., Chen S., Kenswil K.J.G., Mylona M.A., Sanders M.A., Hoogenboezem R.M., Bindels E.M.J., Adisty M.N., Van Strien P.M.H. (2016). Mesenchymal inflammation drives genotoxic stress in hematopoietic stem cells and predicts disease evolution in human pre-leukemia. Cell Stem Cell.

[bib85] Zhao E., Xu H., Wang L., Kryczek I., Wu K., Hu Y., Wang G., Zou W. (2012). Bone marrow and the control of immunity. Cell Mol. Immunol..

[bib86] Zhong L., Yao L., Tower R.J., Wei Y., Miao Z., Park J., Shrestha R., Wang L., Yu W., Holdreith N. (2020). Single cell transcriptomics identifies a unique adipose lineage cell population that regulates bone marrow environment. ELife.

