# Supplementary material for: Deconvolution of the hematopoietic stem cell microenvironment reveals a high degree of specialization and conservation
Source: iScience. 2022 Apr 8;25(5):104225. doi: 10.1016/j.isci.2022.104225 (PMC9046238; doi:10.1016/j.isci.2022.104225)
Supplement: Document S1. Figures S1–S9 [file mmc1.pdf]

iScience, Volume *25*

Supplemental information

Deconvolution of the hematopoietic stem cell microenvironment reveals a high degree of specialization and conservation

Jin Ye, Isabel A. Calvo, Itziar Cenzano, Amaia Vilas, Xabier Martinez-de-Morentin, Miren Lasaga, Diego Alignani, Bruno Paiva, Ana C. Viñado, Patxi San Martin-Uriz, Juan P. Romero, Delia Quilez Agreda, Marta Miñana Barrios, Ignacio Sancho-González, Gabriele Todisco, Luca Malcovati, Nuria Planell, Borja Saez, Jesper N. Tegner, Felipe Prosper, and David Gomez-Cabrero

Supplementary Figures

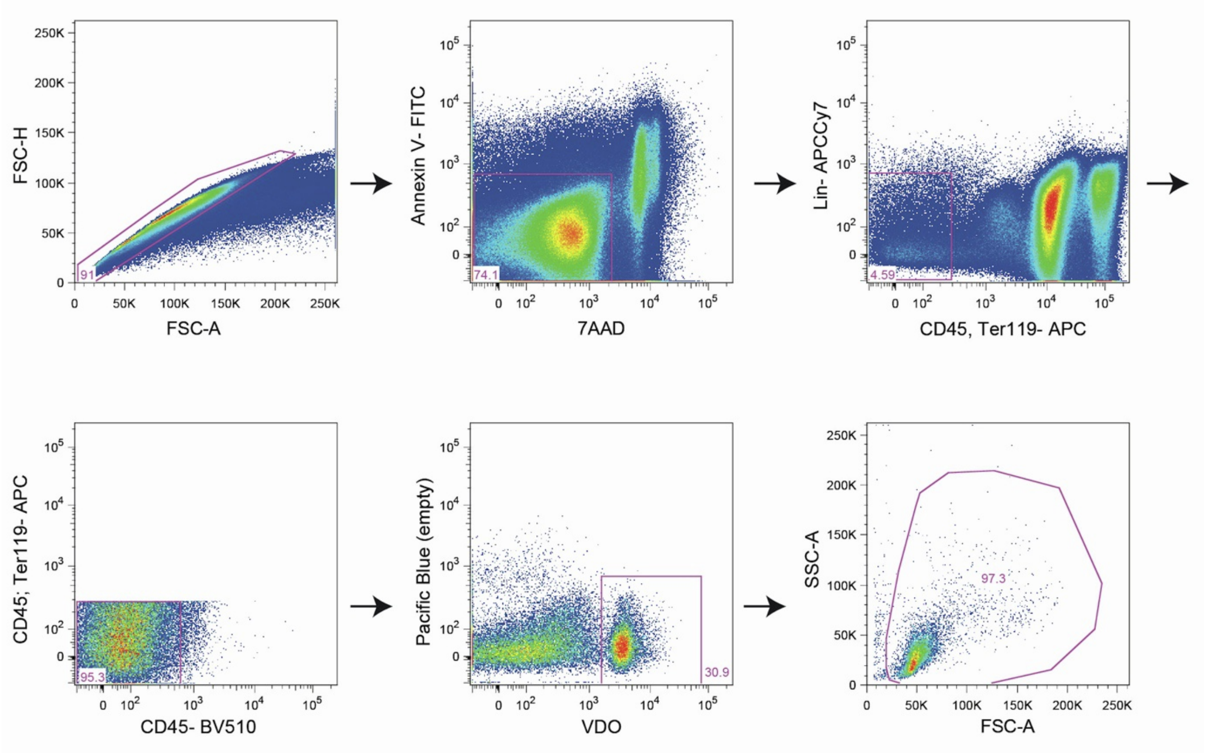

Figure S1. Cell sorting strategy for in-house dataset, related to Figure 1. Cell sorting strategy for isolation of mouse BM microenvironment cells (Lived niche cells: Annexin V^- 7- AAD^- Lin^- $CD45^-$ $Ter119^-$ VDO^+).

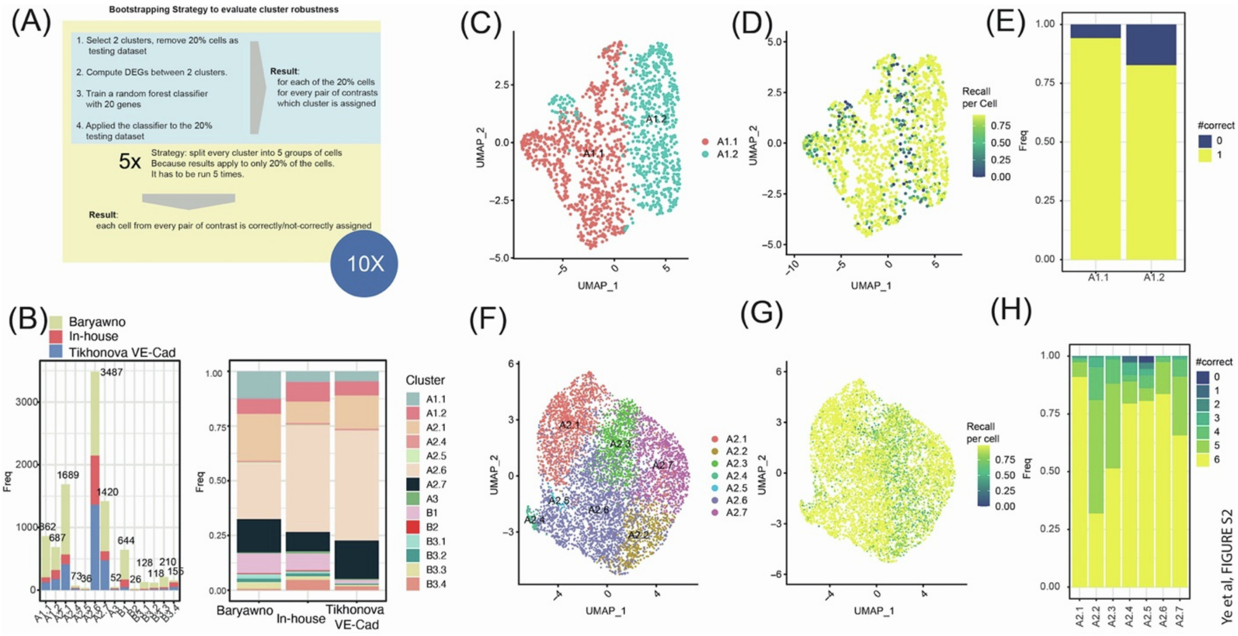

Figure S2. Additional information on the clustering analysis, related to Figure 2. **(A)** Description of the bootstrapping strategy for evaluation cluster robustness (see also Methods). **(B)** Left: representation of the number of cells for each cluster and contribution of each dataset to the final endothelial clusters. Right: proportions of subclusters in each data-set. **(C-E)** Illustration of the sub-clustering, recall per cell and summary of recall per cells for every cluster respectively for the analysis of cluster A1. **(F-H)** Demonstration of the same analysis for cluster A2.

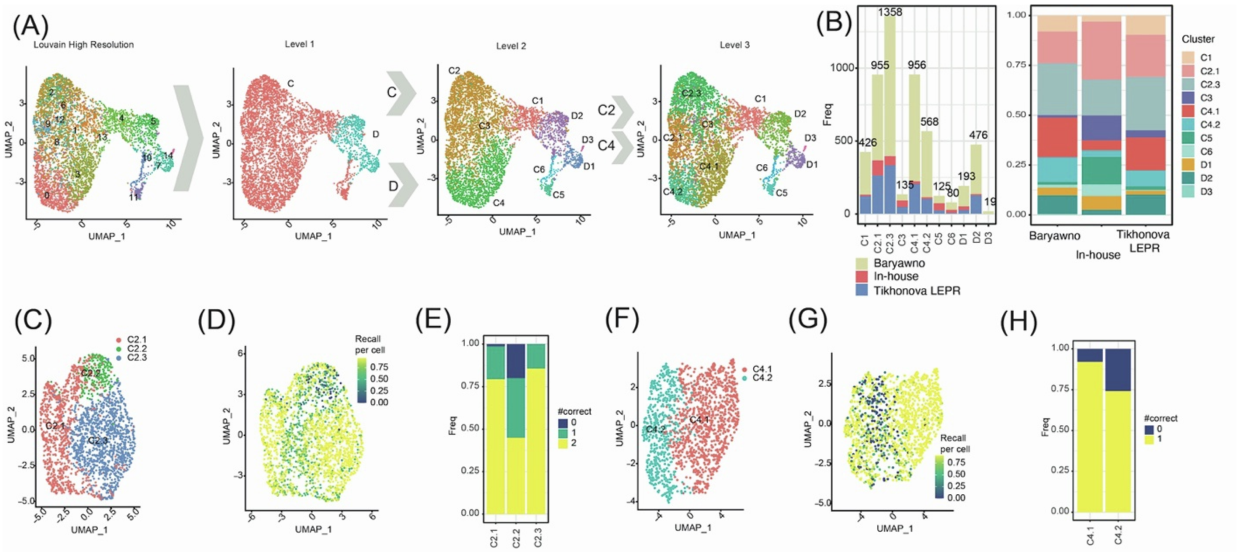

Figure S3. High-resolution clustering strategy of mesenchymal cells, related to Figure 2.
(A) Clustering strategy: Mesenchymal. An upper limit to cluster is set for the clustering (left panel) using Louvain high-resolution clustering. Then, an iterative divide-and-conquer strategy identifies the optimal level of clusters for a given set of cells, and, within each cluster, it evaluates possible sub-clustering. Level 1 (second panel from the left), Level 2 (third panel) and Level 3 (fourth panel) shows the clustering after every iteration. **(B)** Left: representation of the number of cells for each cluster and contribution of each data set to final mesenchymal clusters. Right: proportions of subclusters in each data-set. **(C-E)** Robustness of the analysis for sub-clustering C2 (from Level 2 to Level 3). Specifically: **(C) I**llustration of the identified clusters**; (D)** Depiction of every cell, *how many times it is assigned correctly to a cluster using a random-forest + bootstrapping strategy* (see Methods); **(E)** Summary of the results (d) per cluster. **(F-H)** Demonstration of the same analysis for cluster C4.

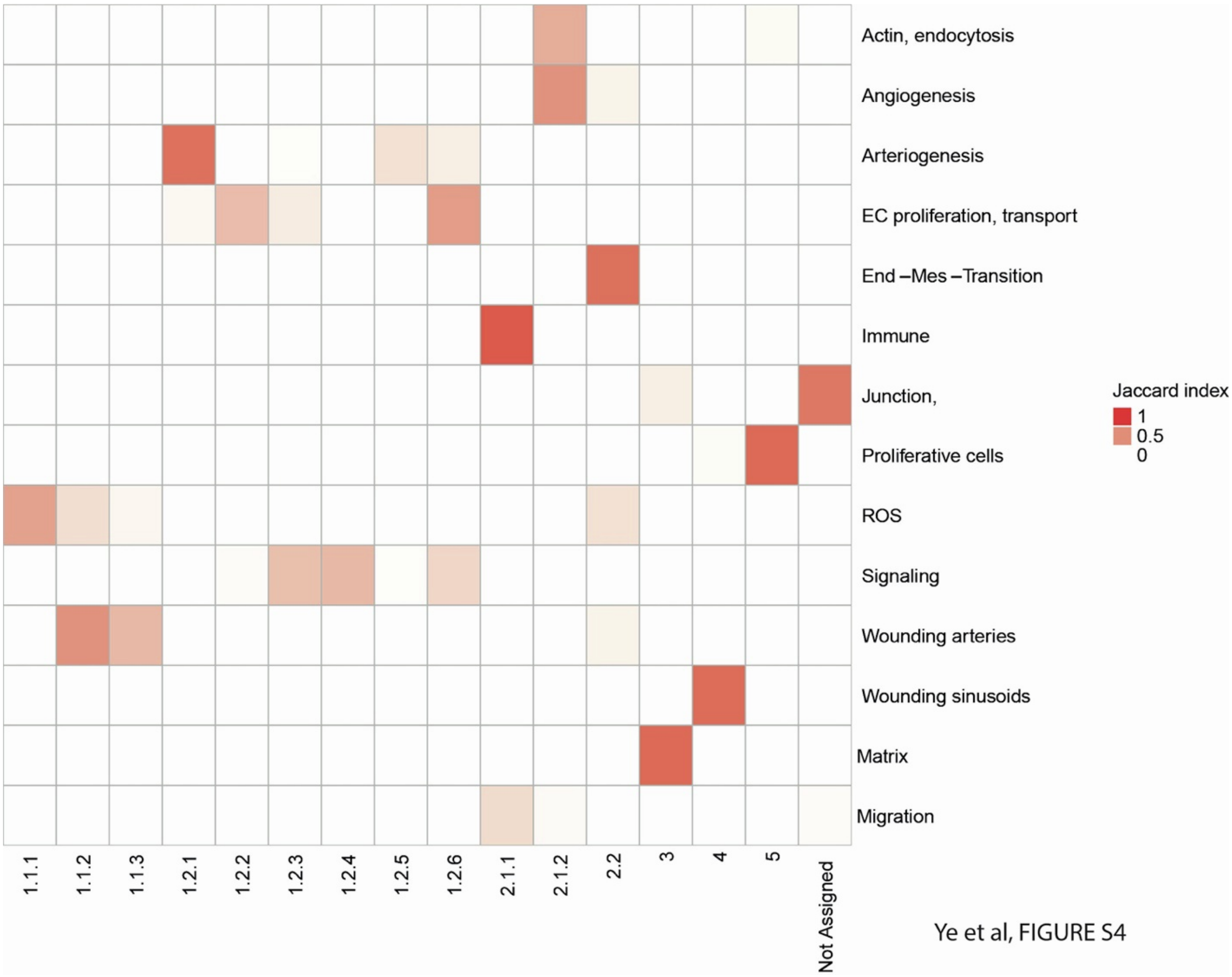

<u>Figure S4. Added value: identification of clusters</u>, related to Figure 3, 4 and 5. Pairwise Jaccard Index analysis between Baryawno dataset only and the identified clusters observed with the integrated dataset (rows).

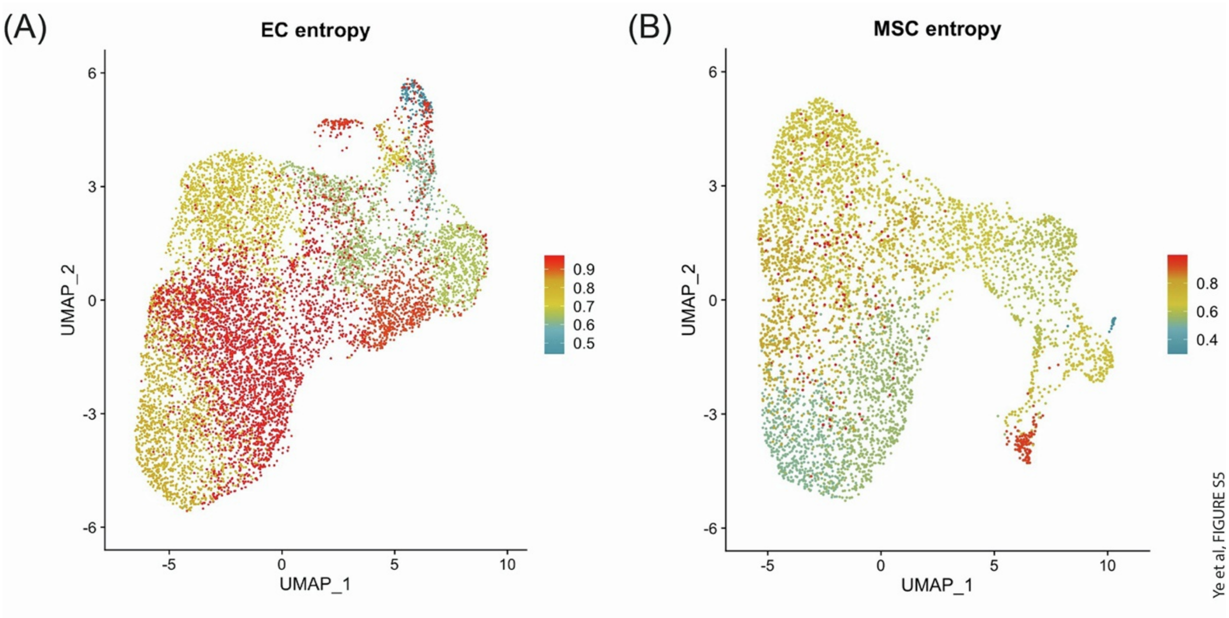

<u>Figure S5</u>. <u>Entropy Analysis</u>, related to Figure 3. **(A)** Entropy analysis of EC cells. **(B)** Entropy analysis of MSC cells.

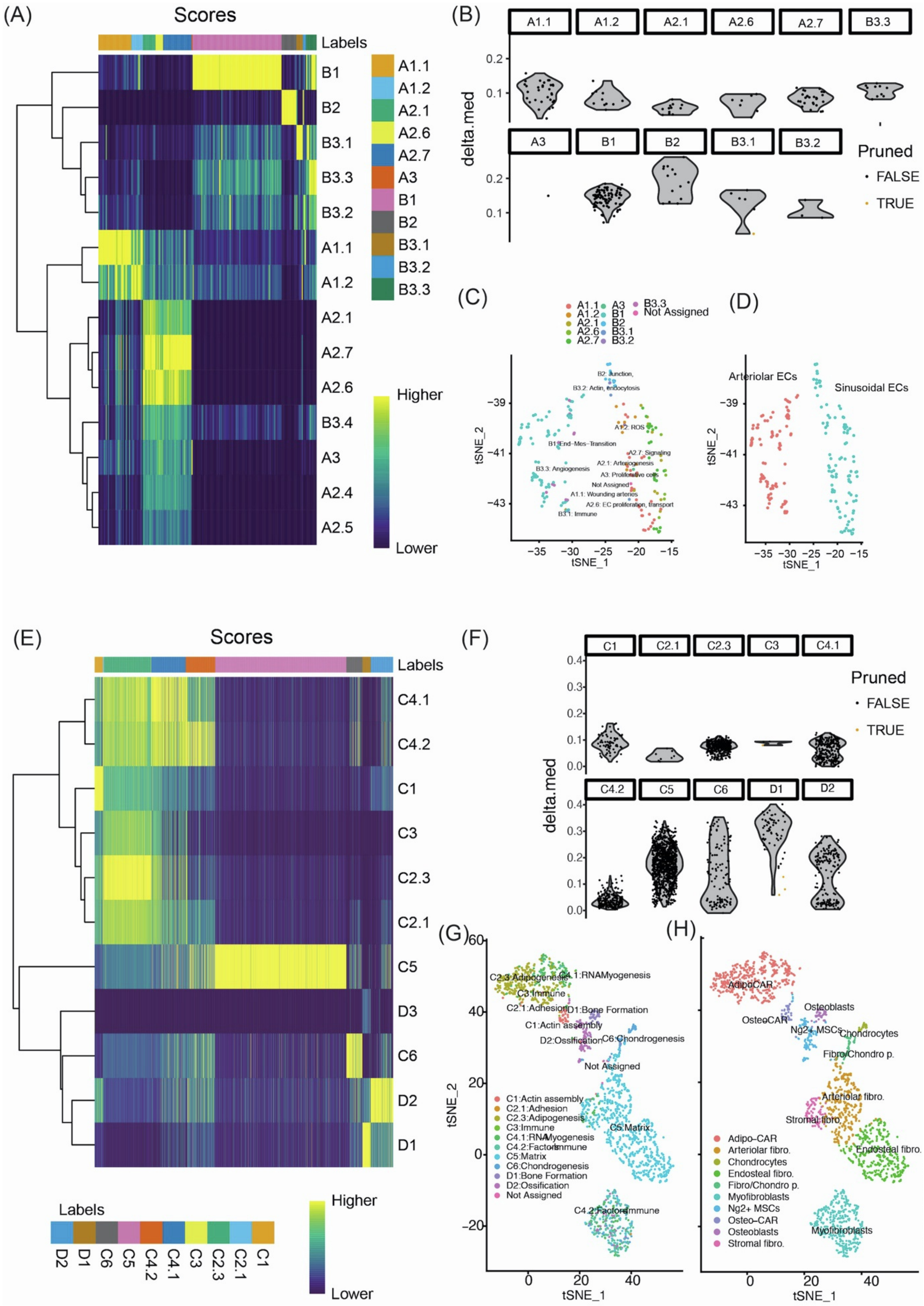

Figure S6. Annotation of Baccin et al, cells. related to Figure 7. **(A)** Heatmap score-derived with the annotations and scores assigned to EC cells. **(B)** EC Score distribution per cluster after cells assigned to clusters based on score. **(C)** t-SNE plot of EC Baccin et al. data-set with assigned annotations. **(D)** t-SNE plot of EC Baccin et al. data-set with original annotations. **(E,F,G,H)** are similar to (A,B,C,D) respectively but considering MSC analysis.

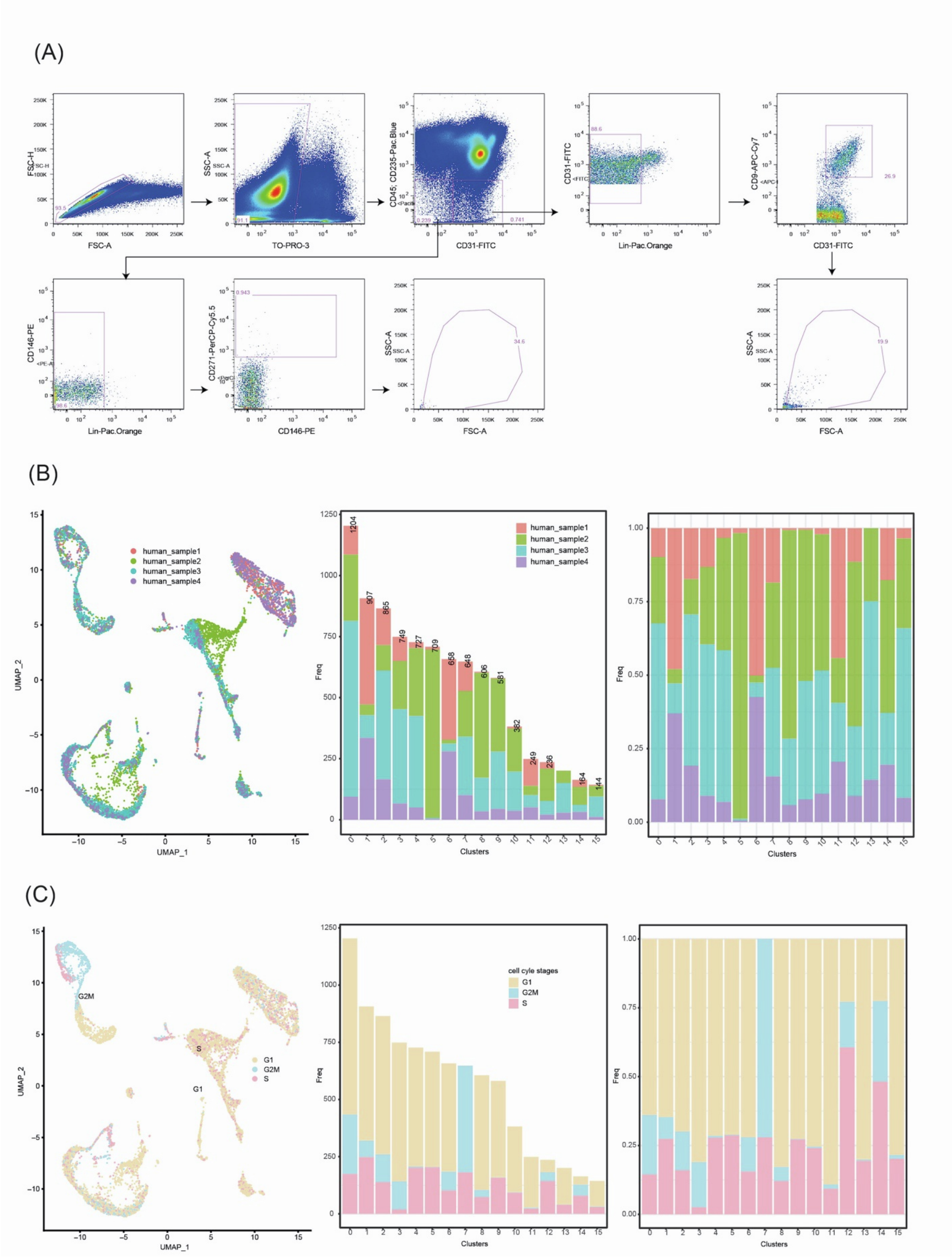

<u>Figure S7. Analysis of human samples</u>, related to Figure 6 **(A)** Cell sorting strategy for isolation of human bone marrow endothelial (TO-PRO-3$^-$, Lin$^-$, CD45$^-$, CD235$^-$, CD9$^+$, CD31$^+$) and mesenchymal-osteolineage (TO-PRO-3$^-$, Lin$^-$, CD45$^-$, CD235$^-$, CD31$^-$, CD271$^+$, CD146$^{+/-}$) cells. **(B)** Left panel: UMAP representation of cells quantified after filtering showing the sample of origin; Middle Panel: number of cells per cluster and sample of origin; Right panel: proportion of cells per cluster and sample of origin. **(C)** Left panel: UMAP visualization of cells quantified after filtering and showing the cell cycle stage; Middle Panel: number of cells per cluster and cell cycle stage; Right panel: proportion of cells per cluster and cell cycle stage.

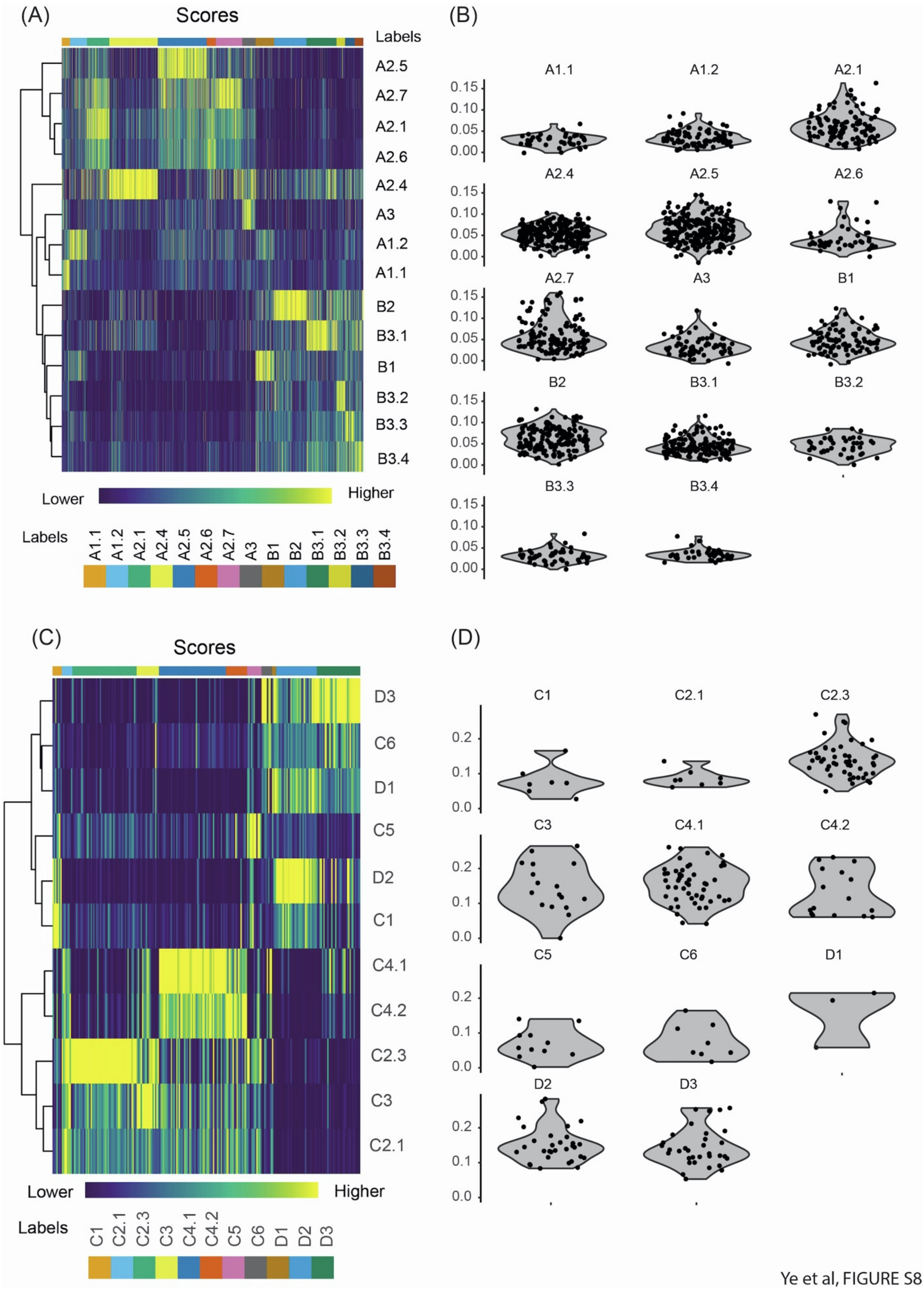

<u>Figure S8. Conservation analysis of the EC and MSC population in the human BM microenvironment</u>, related to Figure 7. Annotation of the human cells using the identified endothelial (**A**) and mesenchymal (**C**) mouse markers through SingleR analysis. Violin plots of the associated scores for endothelial (**B**) and mesenchymal (**D**) subclusters.

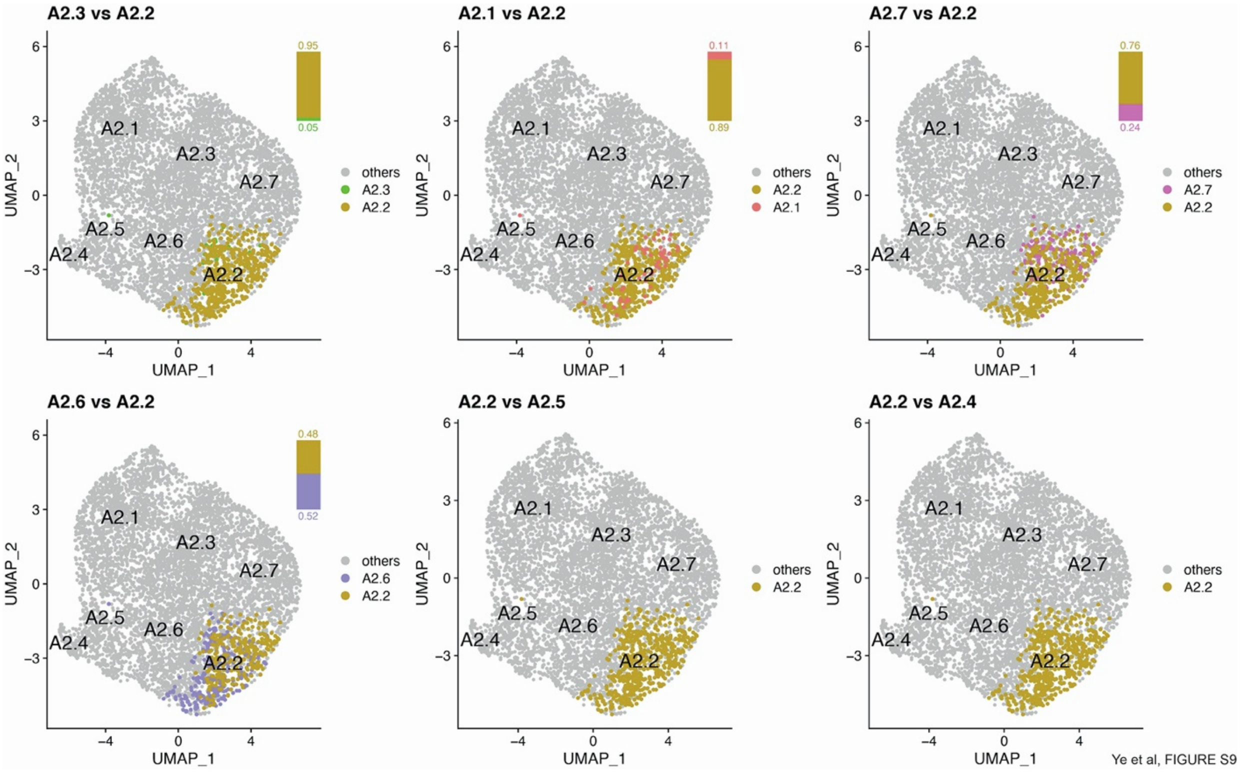

Figure S9. Assignment of cells from non-robust clusters, related to STAR Methods. Example of how cells can be assigned to other clusters once they have been identified as non-robust.
